# Genome-wide association studies of human and rat BMI converge on synapse, epigenome, and hormone signaling networks

**DOI:** 10.1016/j.celrep.2023.112873

**Published:** 2023-07-31

**Authors:** Sarah N. Wright, Brittany S. Leger, Sara Brin Rosenthal, Sophie N. Liu, Tongqiu Jia, Apurva S. Chitre, Oksana Polesskaya, Katie Holl, Jianjun Gao, Riyan Cheng, Angel Garcia Martinez, Anthony George, Alexander F. Gileta, Wenyan Han, Alesa H. Netzley, Christopher P. King, Alexander Lamparelli, Connor Martin, Celine L. St. Pierre, Tengfei Wang, Hannah Bimschleger, Jerry Richards, Keita Ishiwari, Hao Chen, Shelly B. Flagel, Paul Meyer, Terry E. Robinson, Leah C. Solberg Woods, Jason F. Kreisberg, Trey Ideker, Abraham A. Palmer

**Affiliations:** 1 Department of Medicine, University of California San Diego, La Jolla, CA 92093, USA; 2 Program in Bioinformatics and Systems Biology, University of California San Diego, La Jolla, CA 92093, USA; 3 Department of Psychiatry, University of California San Diego, La Jolla, CA 93093, USA; 4 Program in Biomedical Sciences, University of California San Diego, La Jolla, CA 93093, USA; 5 Center for Computational Biology & Bioinformatics, Department of Medicine, University of California, San Diego, La Jolla, CA 92093, USA; 6 Department of Physiology, Medical College of Wisconsin, Milwaukee, WI 53226, USA; 7 Department of Pharmacology, University of Tennessee Health Science Center, Memphis, TN 38163, USA; 8 Clinical and Research Institute on Addictions, University at Buffalo, Buffalo, NY 14203, USA; 9 Department of Human Genetics, University of Chicago, Chicago, IL 60637, USA; 10 Department of Psychiatry, University of Michigan, Ann Arbor, MI 48109, USA; 11 Department of Psychology, University at Buffalo, Buffalo, NY 14260, USA; 12 Department of Genetics, Washington University, St. Louis, MO 63110, USA; 13 Department of Pharmacology and Toxicology, University at Buffalo, Buffalo, NY 14203, USA; 14 Michigan Neuroscience Institute, University of Michigan, Ann Arbor, MI 48109, USA; 15 Department of Psychology, University of Michigan, Ann Arbor, MI 48109, USA; 16 Department of Internal Medicine, Wake Forest School of Medicine, Winston-Salem, NC 27157, USA; 17 Institute for Genomic Medicine, University of California San Diego, La Jolla, CA 92093, USA

## Abstract

A vexing observation in genome-wide association studies (GWASs) is that parallel analyses in different species may not identify orthologous genes. Here, we demonstrate that cross-species translation of GWASs can be greatly improved by an analysis of co-localization within molecular networks. Using body mass index (BMI) as an example, we show that the genes associated with BMI in humans lack significant agreement with those identified in rats. However, the networks interconnecting these genes show substantial overlap, highlighting common mechanisms including synaptic signaling, epigenetic modification, and hormonal regulation. Genetic perturbations within these networks cause abnormal BMI phenotypes in mice, too, supporting their broad conservation across mammals. Other mechanisms appear species specific, including carbohydrate biosynthesis (humans) and glycerolipid metabolism (rodents). Finally, network co-localization also identifies cross-species convergence for height/body length. This study advances a general paradigm for determining whether and how phenotypes measured in model species recapitulate human biology.

## In brief

Wright et al. identify a conserved molecular network underlying body mass index in humans and rats. Genes in this network, which are also associated with body-size phenotypes in mice, describe key processes including neuronal, epigenetic, and hormonal regulation. This work advances a general paradigm for cross-species GWAS translation.

## Graphical Abstract

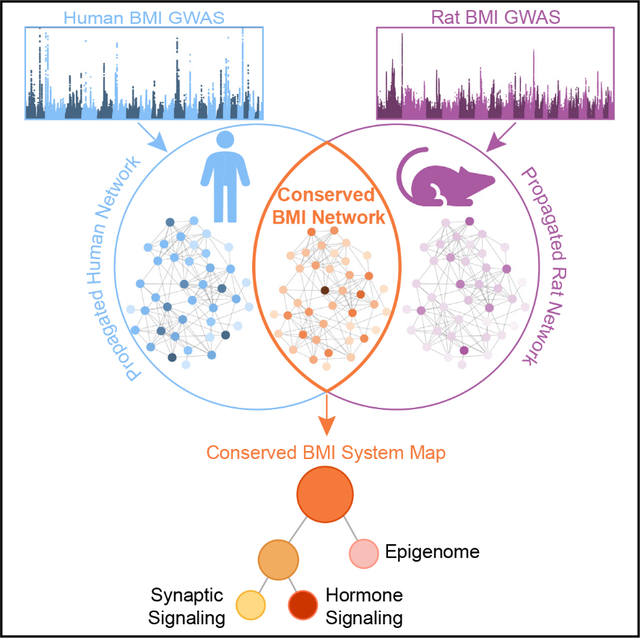

## INTRODUCTION

Rodents and other model organisms provide unique advantages for investigating the molecular basis of human traits and diseases. However, the widespread assumption that disease biology is conserved across mammals is primarily based on conjecture and anecdotal evidence and remains controversial.^1–3^ Methods to rigorously assess the validity of animal models are urgently needed, particularly for complex polygenic traits. One such trait is body mass index (BMI), which is used to diagnose obesity, a condition with a large and growing disease burden worldwide.

Complex traits are commonly examined by genome-wide association studies (GWASs), which have identified thousands of genetic loci underlying numerous diseases. Despite this success, synthesizing the numerous loci identified by GWASs into biologically interpretable results remains challenging.^4,5^ By design, the hypothesis-free nature of GWASs lends impartiality; on the other hand, knowledge about the functional relationships between genes has enormous potential to improve sensitivity and interpretability.

Model organism data are commonly used to provide a more holistic interpretation of GWAS results.^6^ For example, single-gene knockouts can address ambiguity about which variants are causal at a given locus (i.e., the fine-mapping problem), and integration of model organism omics datasets can clarify causal biological mechanisms.^7,8^ However, such approaches do not capture the polygenic architecture of complex traits. An attractive alternative is to perform GWASs in heterogeneous model organism populations, which is a complementary approach that recapitulates the polygenic nature of complex human traits. With this motivation, many GWASs have been performed in rats,^9–12^ mice,^13–16^ fruit flies,^17–19^ and other species.^20–23^

In cases where similar traits have been studied via GWASs in both humans and model organisms, it is possible to ask whether the results implicate orthologous genes. Thus far, the concordance of genes implicated by separate GWASs in two species has typically been limited by incomplete power, differences in the variants that are common in each population, and many other factors. Furthermore, the most functionally similar gene in a model species can be a family or pathway member rather than the most sequence-similar ortholog.^24^

One means of coping with these challenges is to leverage biological knowledge networks. Biological networks provide prior information on the interactions among genes and, therefore, aid the translation of implicated genes’ mechanistic insights into relevant cellular processes, cell types, tissues, and dynamics.^25–27^ We and others have shown that integrating genetic data with biological knowledge networks can improve the interpretability of findings,^28–31^ including via studies in model organisms.^32,33^ We recently expanded on these approaches by developing a network “co-localization” framework (NetColoc), which evaluates the convergence of two gene sets within a biological network. This approach was then used to identify convergent genetic circuits underlying autism spectrum disorder and congenital heart disease, which are comorbid.^34,35^

**Figure 1.**
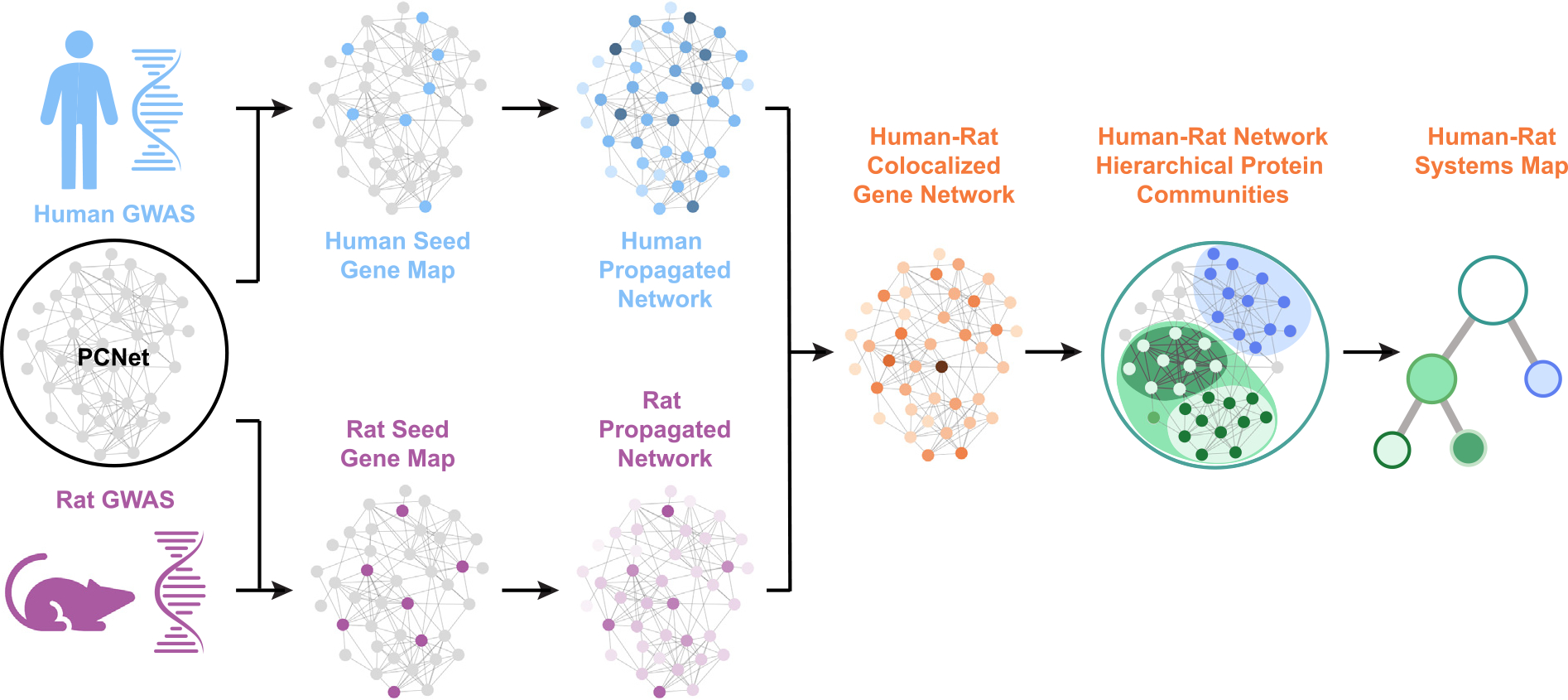
Generation of a cross-species conserved BMI systems map GWAS results for human BMI (top, blue) and rat BMl (bottom, purple) are independently propagated through a molecular interaction network (PCNet) to measure the proximity of all genes to the input sets. Colocalized genes are identified as genes that are proximal in the network to BMI seed genes in both species (orange). Within this subset, nested communities of densely connected genes are identified to generate a hierarchy of systems representing the shared biology of BMI across humans and rats. A toy network is shown for illustrative purposes.

Here, we explore the network co-localization approach for cross-species translation of GWAS results (Figure 1). As proof of concept, we use this approach to study BMI, a highly polygenic and medically important trait that has been extensively studied in both humans and rodent models.^36,37^ We show that, while the specific genes identified by human and rat GWASs do not show significant overlap, they converge on a conserved molecular interaction network. This conserved network provides insight into the shared etiology of BMI across mammals, and our approach establishes a general paradigm for assessing the extent to which an animal model does, or does not, recapitulate human biology.

## RESULTS

### GWASs associate different genes with BMI in humans and rats

**Figure 2.**
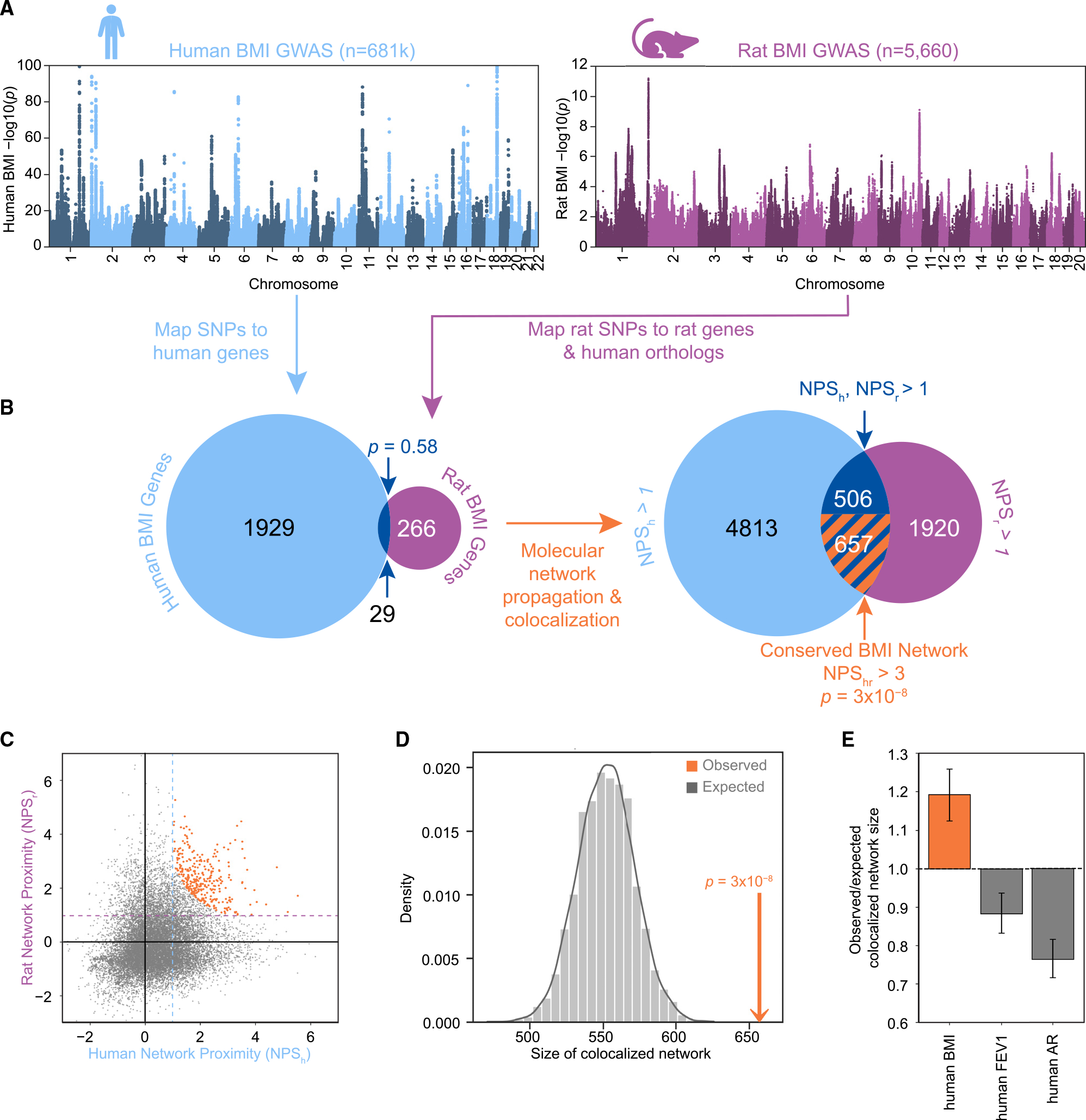
Convergence of rat and human BMI GWAS results at the gene and network level (A) GWAS Manhattan plots for human BMI (left, light blue) with a maximum displayed log(p) = 100 and rat BMI (right, purple) with a maximum displayed −log(p) = 12. (B) Left, Venn diagram representing the gene-level overlap of GWAS results after mapping to human genes; dark blue represents the 29 orthologous genes implicated in both species. Right, Venn diagram of genes passing network proximity score (NPS) thresholds after network co-localization. NPS_h_ > 1 (light blue); NPS_r_ > 1 (purple); NPS_h_ > 1 and NPS_r_ > 1 (dark blue); NPS_h_ > 1, NPS_r_ > 1, and NPS_hr_ > 3 (conserved network, orange stripes). Overlap p values were calculated via hypergeometric test. (C) Human and rat NPS for all non-seed genes, with genes passing all thresholds for the conserved BMI network (NPS_hr_ >3, NPS_h_ >1, and NPS_r_ > 1) shown in orange. Dotted horizontal and vertical lines indicate the single-species thresholds. (D) Observed (orange arrow) versus expected (gray distribution) size of the conserved BMI network following 10,000 permutations of NPS_r_ labels, with p value calculated via *Z* test. (E) The observed-to-expected ratio of colocalized network size for rat BMI with human BMI (left orange bar) and two negative control comparisons (gray). FEV1,forced expiratory volume per second; AR, allergic rhinitis symptoms. Vertical bars indicate 95% confidence intervals. See also Figure S2 and Table S1.

We obtained genome-wide significant loci from a recent large human GWAS meta-analysis for BMI^38^ (n = 681,275; Figure 2A). Positional mapping of these loci to the human genome identified 1,958 significant BMI-associated genes at a Bonferroni-corrected threshold of p < 2.5 × 10^−6^. In addition, we performed a separate GWAS for BMI in outbred heterogeneous stock (HS) rats, using data from a previous study,^37^ together with an additional 2,487 previously unreported HS rats to obtain a single, outbred population (n = 5,660; Figure 2A). Outbred HS rats have larger linkage disequilibrium (LD) blocks compared to humans,^39^ meaning that a GWAS represents a smaller number of independent tests. Therefore, we used a relaxed significance threshold (p < 1.0 × 10^−4^) for the rat GWAS, identifying 476 BMI-associated rat genes by positional mapping. Of these, 295 had unambiguous human orthologs (Figure S1). We define the significant BMI gene sets for humans and rats as “seed” genes and assume that both sets represent a mixture of true and false positives. Overall, we found 29 genes in common between the seed genes for humans and rats, a number that was not significantly different than expected by chance for gene sets of this size (Figure 2B; p = 0.58, hypergeometric test).

### A conserved molecular network underlies BMI across species

We next examined the molecular pathways in which the BMI genes function. For this purpose, we used the Parsimonious Composite Network (PCNet), a resource of ~2.7 million pairwise associations among human genes.^40^ PCNet is formulated from a consensus of 21 physical and functional interaction databases and integrates multiple lines of evidence, including protein-protein interactions, mRNA and protein co-expression across tissues, literature curation, and other measures. We assigned a human BMI network proximity score (NPS_h_) to each gene using a random walk algorithm^41^ to compute the normalized number of steps through the network to reach that gene from the seed set of 1,958 human BMI genes. We repeated this process using the 295 human orthologs of the rat BMI seed genes, resulting in a rat BMI network proximity score (NPS_r_) for each gene. Finally, we calculated the product of the two proximity scores to compute NPS_hr_ = NPS_h_ × NPS_r_ (Table S1). In this way, genes with the highest NPS_hr_ tended to be close in the molecular network to BMI seed genes from both species, even if they were not seed genes in either species.

We found that NPS_hr_ values were significantly higher than expected for permuted sets of human and rat genes (p = 1 × 10^−3^; Figure S2A). We chose to threshold this score (NPS_hr_ > 3 and NPS_h_ and NPS_r_ each >1; Figures 2B and 2C) to create a highly colocalized subnetwork of PCNet that we called the “conserved BMI network.” This network included 657 genes, which was significantly more than expected by chance (Figure 2D, p = 3 × 10^−8^). Thus, although the two BMI GWASs identified different genes in humans and rats, the molecular networks encoded by those genes show significant convergence. The 657-gene conserved BMI network covered 21 genes that were both human and rat seeds, with an additional 207 human seed genes and 92 rat seed genes. These genes represented the most interconnected genes from the input sets. The remaining 337 conserved network genes were not directly identified by either the human or the rat GWAS but were instead implicated via network proximity.

To explore whether network convergence extends to other traits, we performed a similar network analysis of human height and its rodent analog, rat body length. These traits have been widely phenotyped in both species and are used to calculate BMI, so they are partially but not completely correlated with BMI.^42^ Unlike BMI, the GWAS for human height and the GWAS for rat body length identified a significant number of orthologous genes (p = 6 × 10^−11^; Figure S2B). However, consistent with our BMI results, we found that the molecular networks defined by human height genes and rat body length genes show substantially increased agreement, even when controlling for the number of shared genes (p = 3 × 10^−20^; Figures S2C and S2D; STAR Methods).

We also examined the network co-localization of rat BMI genes with human GWAS results for four negative control traits that had SNP heritability similar to human BMI (0.15 < *h*^*2*^ < 0.35) but were not genetically correlated with BMI or height (genetic correlation |*r*_g_| < 0.1): allergic rhinitis symptoms, forced expiratory volume per second, smoking status (never smoked cigarettes), and presence of non-cancerous neoplasms. The rat BMI genes did not show significant network convergence with the genes identified by the negative control traits (Figures 2E and S2E). Because PCNet is a human interaction network, we also tested the network co-localization of the human and rat BMI seed genes within a molecular network consisting of 16,787 rat genes and 277,852 high-confidence rat interactions sourced from the STRING database.^43^ We observed comparable network conservation with this rat network (Figure S2E; p = 2 × 10^−4^; STAR Methods).

### The conserved BMI network implicates distinct gene communities and functions

**Figure 3.**
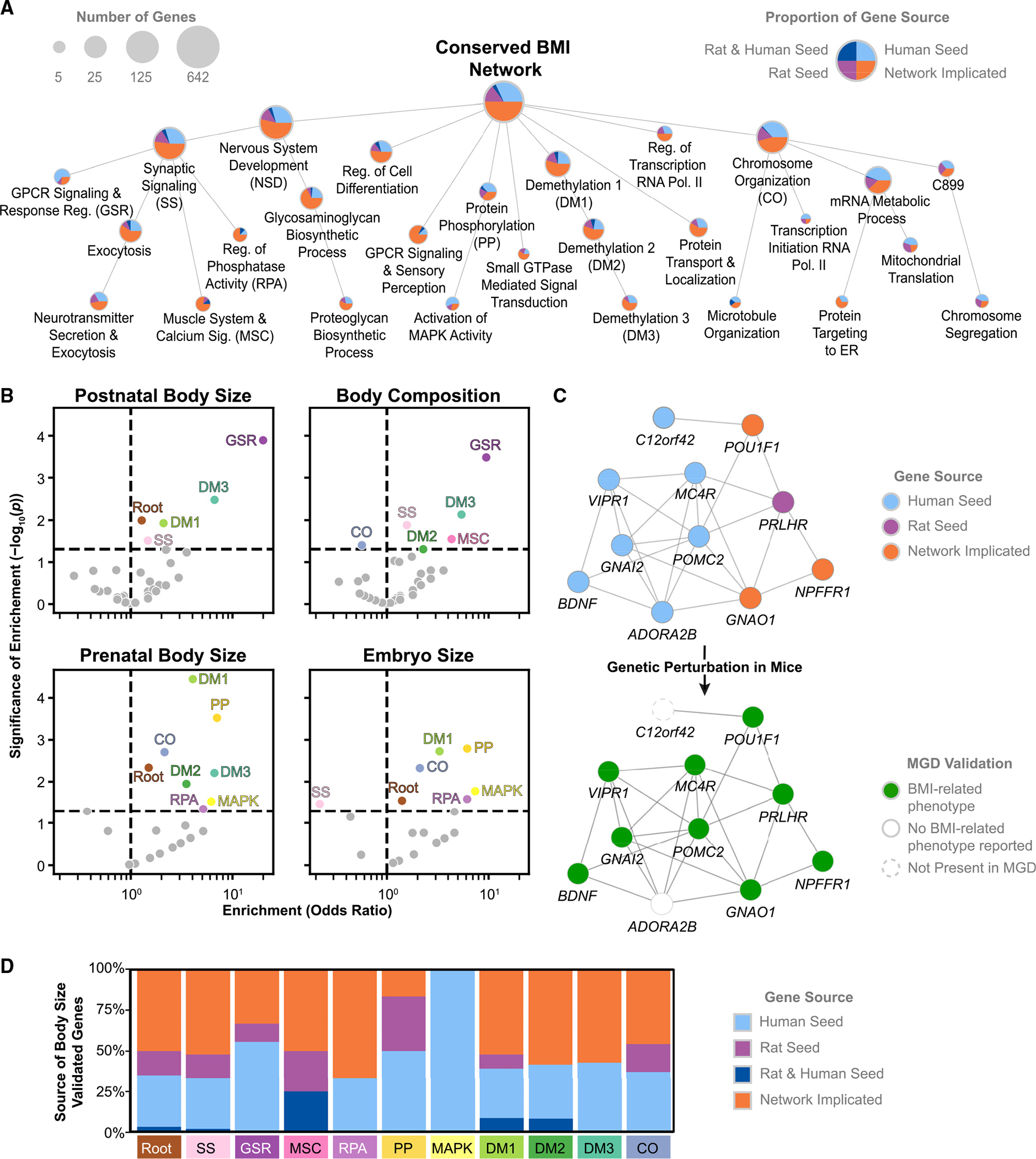
The conserved BMI systems map (A) The conserved BMI network as a hierarchy of interacting gene communities. Nodes represent individual gene communities, with size indicating the number of genes (minimum five, maximum 642). Edges indicate smaller communities that are nested within larger communities. Pie charts indicate the fraction of community genes that are human seed genes, rat seed genes, seed genes in both species, or network-implicated genes. Systems are annotated based on significantly associated Gene Ontology (GO) Biological Process terms. Only annotated communities and those necessary to connect annotated communities are shown. (B) Enrichment of each annotated community in (A) for genes associated with four BMI-related phenotypes (defined as postnatal body size, prenatal body size, embryo size, and body composition) in the Mouse Genome Database (MGD). Colored points show the nominally significantly enriched communities (Fisher’s exact test), with all other communities in light gray. (C) The GPCR signaling and response regulation community (GSR) subnetwork. Node color indicates the source of genes in the subnetwork (top) and associations with any of the four BMI-related phenotypes in mice (bottom). (D) Sources of all MGD-validated genes for communities significantly enriched for body-size-associated genes in (B). MGD-validated genes were defined as genes linked to at least one BMI-related phenotype in MGD. See also Figures S3, S6A, and Table S1.

We observed that genes in the conserved BMI network were not uniformly connected but were organized within densely interacting communities (Figure S3A). To identify these, we used multiscale community detection, which yielded a hierarchical map of 61 gene communities, which we term the “BMI systems map” (Figure S3B). This map contained the 642 genes (98%) that were part of the conserved BMI network’s largest connected component. We found that 27 communities in this map could be confidently annotated with enriched gene functions (Figure 3A) and that these functions were more numerous and specific than those identified for the entire conserved network or for sets of seed genes alone. We observed 331 significant annotations after filtering, compared to 45 for the conserved network, 59 for the human seed genes, and zero for the rat seed genes. Many of the annotations identified for the seed genes were captured via the annotated BMI systems map, particularly those relating to synaptic signaling and cellular morphogenesis. However, the hierarchical systems map identified additional and more specific functions, such as catecholamine synthesis and transport, demethylation, and monoamine transport (Table S2). The largest branch of the BMI systems map (by the number of genes covered) centered on nervous system development and physiology, with distinct sub-communities including G-protein-coupled receptor (GPCR) signaling, neurotransmitter secretion, and muscle development and function. A second major branch concerned nucleic acid and chromosomal processes, including mRNA transcription and protein translation. The remaining branches included functions such as chromatin regulation via demethylation, cell differentiation, and communities of mitogen-activated protein (MAP) kinases.

To validate this BMI systems map, we consulted the extensive catalog of mouse mutation experiments maintained by the Mouse Genome Database^44^ (MGD). MGD records nearly 364,000 various phenotypic changes after whole-body or tissue-specific genetic perturbations in mice, which we used to assess the functional role of genes within the BMI systems map. Focusing on body size and composition phenotypes, we found that mouse orthologs of 39% of genes within the conserved BMI network had been associated with at least one body-size phenotype in mice. We observed that these validated genes were concentrated within particular branches of the BMI systems map, spanning 14 distinct communities in which disruption to community genes leads to MGD phenotypes related to body size or composition in mice (Figure 3B). Within these branches, we observed stronger enrichments for smaller, more specific systems, and, across the hierarchy, we found differentiation between communities associated with postnatal and prenatal body-size phenotypes.

The strongest enrichment for postnatal body size and composition in mice was observed for genes in the GPCR signaling and response regulation community (GSR, n = 11 genes), where disruptions to nine genes led to BMI-related phenotypes in mice (Figure 3C). The MGD-validated genes included some that have been extensively studied in the context of BMI, such as genes for brain-derived neurotrophic factor *BDNF*, melanocortin receptor *MC4R*, and the hormone precursor proopiomelanocortin *POMC*. While these genes were identified in the human BMI GWAS, they lacked equivalent associations in the rat GWAS. In contrast, the MGD-validated prolactin-releasing hormone receptor (PRLHR) was not associated with human BMI, but its rat ortholog (*Prlhr*) was associated with BMI in the rat GWAS (p = 1 × 10^−12^). Beyond seed genes, we observed three network-implicated genes that were validated in MGD. For example, neither *NPFFR1* nor its rat ortholog *Npffr1* were directly identified by their respective GWAS, but *NPFFR1* has been associated with insulin-like growth factor 1 (IGF-1) levels in humans,^45^ and constitutive inactivation of *Npffr1* has been associated with increased susceptibility to weight loss in mice.^46^ As with the GSR community, we observed that network-implicated genes substantially contributed to body-size enrichments throughout the BMI systems map, illustrating the power of our network approach to connect and expand the results of human and rat BMI GWASs (Figures 3D and S3C).

**Figure 4.**
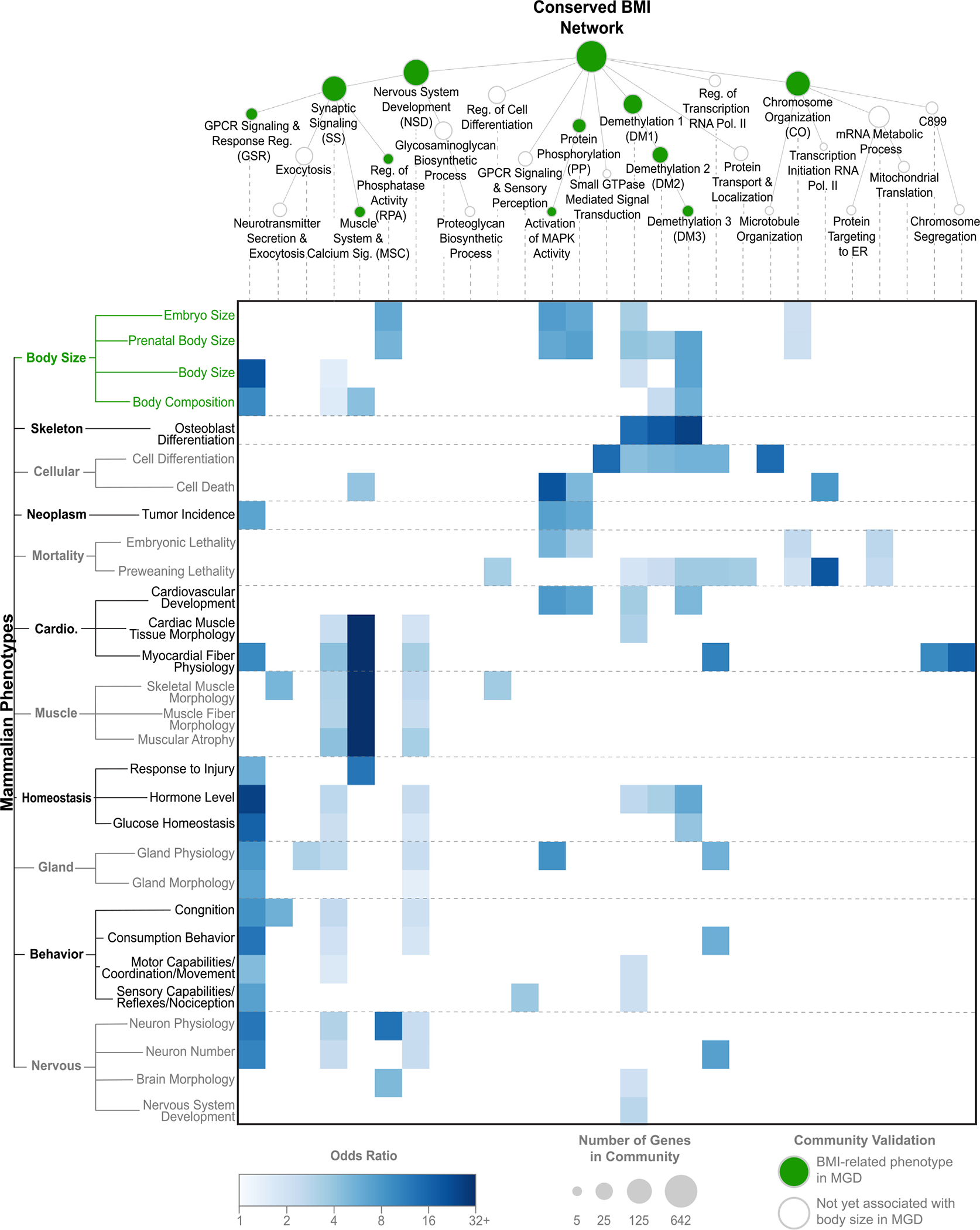
Functional effects of perturbations to conserved BMI systems in mice Reproduction of the conserved BMI systems map (top) with gene communities significantly enriched for mouse BMI-related traits in green (p < 0.05, Fisher’s exact test). Heatmap (bottom) shows the enrichment of communities within the system map for body-size and non-body-size traits in mice, selected to capture the spectrum of phenotypes affected by the systems map (STAR Methods). The OR is shown for any community-phenotype associations that were nominally significant (p < 0.05) via a Fisher’s exact test. Phenotypes are grouped based on the Mammalian Phenotype Ontology (left). See also Table S1.

In addition to body-size phenotypes, genetic disruptions in specific communities were enriched for related tissue and behavioral phenotypes (Figure 4). For example, disruptions to genes in the GSR community led to changes in mouse hormone levels (odds ratio [OR] = 25, p = 3 × 10^−7^), glucose homeostasis (OR = 16, p = 8 × 10^−6^), consumption behavior (OR = 13, p = 5 × 10^−5^), neuron physiology (OR = 13, p = 6 × 10^−5^), and other relevant mouse traits. Given that these same disruptions also affect postnatal body size and composition, the GSR community provides a conserved pathway by which diverse genetic alterations cause key hormone and behavioral changes that, in turn, give rise to gross morphological changes affecting overall body size and mass.

**Figure 5.**
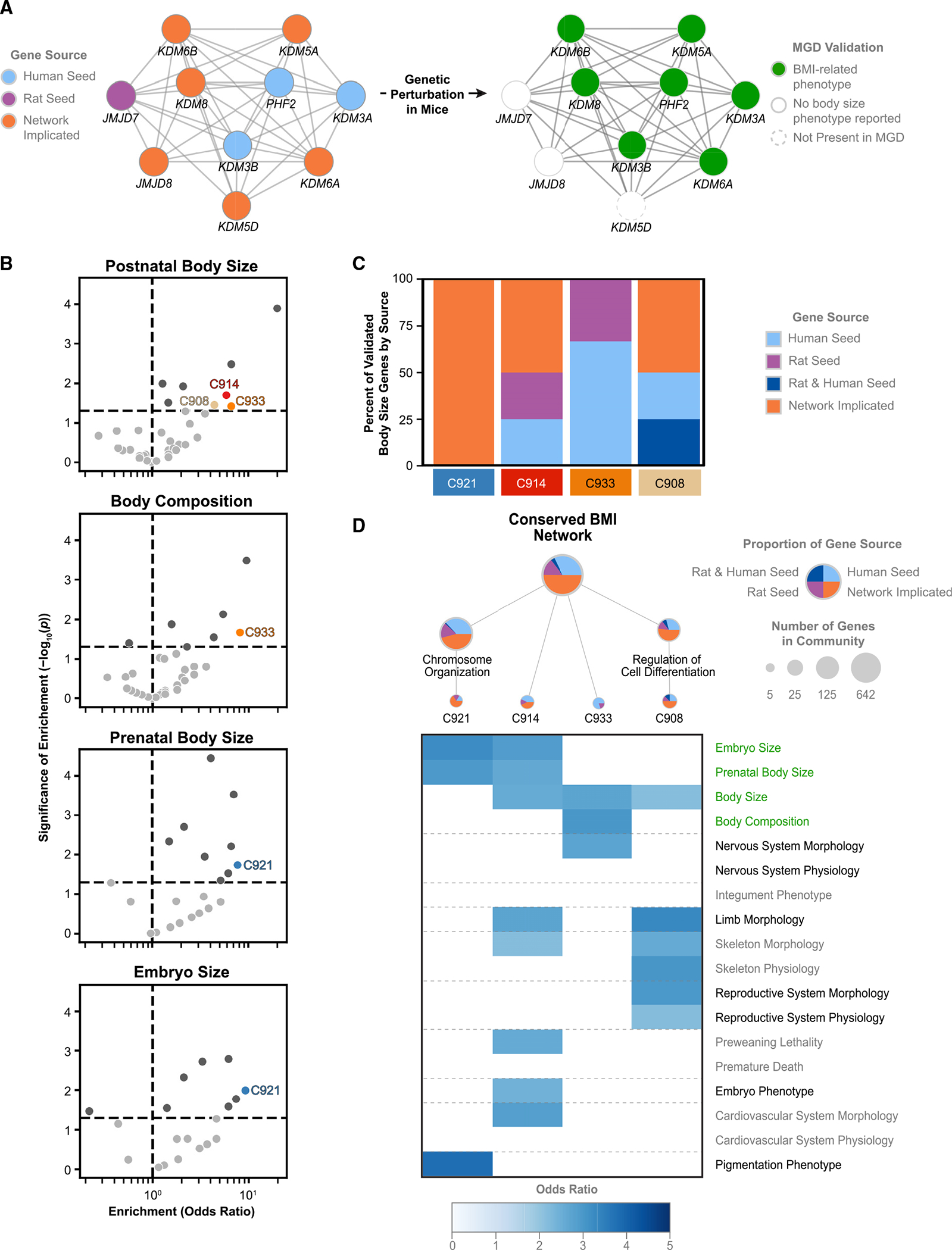
Investigation of gene communities within the conserved BMI systems map (A) Subnetwork of genes in demethylation community 3 (DM3). Node color indicates the source of the genes (left) and associations with any reported BMI-related abnormal phenotypes (postnatal body size, prenatal body size, embryo size, or body composition) in MGD (right). (B) Enrichment of systems map communities for genes associated with the four BMI-related traits in mice via MGD. Colored points highlight significant enrichments (Fisher’s exact test) for unannotated communities. Light gray indicates unannotated communities that are not significantly enriched, and dark gray indicates annotated communities with significant enrichment, as shown in Figure 3B. (C) Sources of all MGD-validated genes for communities significantly enriched for body-size-associated genes in (B). MGD-validated genes were defined as genes linked to at least one BMI-related phenotype in MGD. (D) Unannotated communities within the conserved BMI systems hierarchy (top) with enrichments for selected phenotypes in mice (heatmap, bottom). Odds ratios for all nominally significant (p < 0.05, Fisher’s exact test) enrichments are shown. See also Figures S3, S6A, and Table S1.

Other notable BMI systems included muscle system and calcium signaling (MSC; Figures 4 and S4A), in which genetic perturbations affect muscle morphology leading to abnormal body size and composition, as well as chromatin regulation via demethylation (DM) communities (Figure S4B), in which mutations cause defects in skeletal and cellular differentiation and changes to hormone levels. Notably, at least 50% of the MGD-validated genes in these communities were implicated by the network analysis rather than by the initial human and rat GWASs. For example, the MSC community successfully recovered the X chromosome gene *DMD*, named for its role in Duchenne muscular dystrophy, a condition with a high prevalence of comorbid obesity.^47^ *DMD* and *Dmd* were not identified by either the human or rat GWAS due to the exclusion of sex chromosomes, but *DMD* has been previously linked to BMI in humans and weight changes in mice.^48–50^ The core DM community (DM3) included distinct demethylase genes identified by the human and rat GWASs, as well as four network-implicated demethylases (KDM5A, KDM6A, KDM6B, and KDM8), which have been linked to body-size phenotypes in mice^51–56^ (Figure 5A).

Finally, we observed four communities that did not have clear associations with known biological functions but showed enrichment for genes associated with BMI-relevant phenotypes in mice (Figure 5B); these communities had contributions from both seed and network-implicated genes (Figures 5C and S3C). For example, the C914 community (n = 7; Figure S4C) contained seed genes axin-1 (*AXIN1*) and hedgehog acyltransferase (*HHAT*), as well as network-implicated genes plectin (*PLEC*) and the SKI-like proto-oncogene (*SKIL*). Disruptions to their mouse orthologs *Axin1*, *Hhat, Plec*, and *Skil* led to changes in prenatal and postnatal body size, and the gene community was linked to developmental effects on skeletal, nervous, and cardiovascular system morphology in mice (Figure 5D). We observed further impacts on skeletal and limb morphology in mice via disruptions to genes in community C908 (n = 8; Figure S4D), which was nested within a larger community involved in regulating cellular differentiation. C908 includes four SOX transcription factors: two seed genes (*SOX5, SOX6*) and two network-implicated genes (*SOX3, SOX8*). SOX transcription factors have been associated with hyperinsulinemia in obese mice.^57,58^ Increased expression of *SOX6* may predispose individuals to obesity through the promotion of adipogenesis,^59^ and *Sox8*-deficient mice have significant weight reduction in adulthood due to adipose tissue degeneration.

### Divergent BMI mechanisms across species

**Figure 6.**
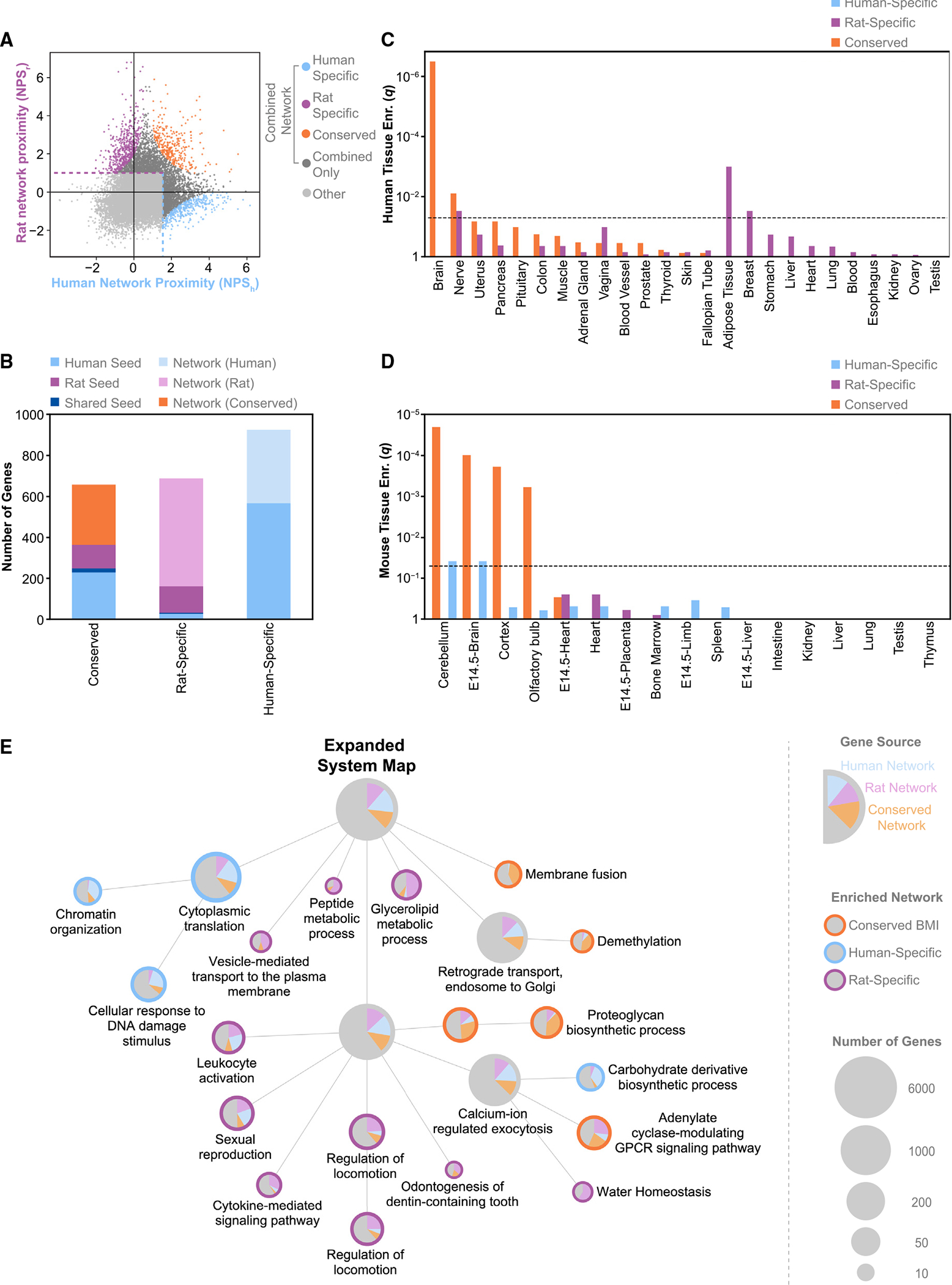
Identification of divergent processes underlying BMI in humans and rats (A) Definition of species-specific subnetworks from NPS_h_ and NPS_r_ showing genes that were not seed genes in either species. Cutoffs for the expanded network are indicated with dotted lines (NPS_h_, blue; NPS_r_, purple). The expanded network includes all nodes in the human-specific, rat-specific, and conserved network, as well as all dark gray nodes; all other nodes are indicated in light gray. Networks are defined as human specific (NPS_h_ > 1.5, NPS_r_ < 0, NPS_h_(NPS_r_−1) < −4), rat specific (NPS_h_ < 1, NPS_r_ > 1, NPS_r_(NPS_h_−1) < −2), or conserved (NPS_h_ > 1, NPS_r_ > 1, NPS_hr_ > 3). (B) The number of genes in the conserved and species-specific subnetworks (x axis) colored by the source of the genes. (C) Tissue enrichment for species-specific and conserved subnetwork genes in human GTEx data, with p values false discovery rate (FDR) corrected (STAR Methods). (D) Tissue enrichment for species-specific and conserved subnetwork genes in mouse ENCODE data, with p values FDR corrected (STAR Methods). (E) Subset of the expanded systems map containing communities enriched for species-specific or conserved genes (q < 0.05, hypergeometric test). Pie charts show the fraction of genes from the conserved and species-specific networks. Systems are annotated based on associated GO Biological Process terms. See also Figures S5, S6, and Table S1.

Apart from the conserved BMI network, we also identified non-overlapping BMI networks distinct to each species. We used the previously calculated NPS_h_ and NPS_r_ values to find genes highly proximal to seed genes of one species but not the other (Figures 6A and S5), yielding human-specific (n = 925 genes) and rat-specific (n = 688 genes) networks (Figure 6B). While the conserved BMI network was significantly enriched for genes expressed in human and mouse brain tissue (Figures 6C and 6D), no brain specificity was observed for the species-specific networks, indicating that these networks are functionally distinct from the conserved BMI network. The rat-specific network was most strongly enriched for orthologs expressed in human adipose tissue (q = 1 × 10^−3^), while the human-specific network showed little tissue specificity across human and mouse tissues. In contrast, the human-specific network contained a significant number of genes associated with body-size phenotypes in mice, while the rat-specific network did not (p_human_ = 0.02, p_rat_ = 0.82, Figure S6A). The MGD-validation rate for the human-specific network was comparable to the conserved network (p = 0.02), which had the highest validation rates for human and rat seed genes, then rat seed genes, then network-implicated genes (Figure S6A).

To investigate potential species-specific BMI-mediating pathways, we created an expanded cross-species BMI network, including all genes with high proximity to human or rat GWAS seed genes (Figure 6A). We then formed an expanded systems map from this network (Figure S6B) and identified communities with an over-representation of conserved, rat-specific, or human-specific genes (Figure 6E). In this way, we highlighted functional systems influencing BMI that are affected by common genetic variation in a species-specific manner. For rats, we found 12 communities containing a significant number of rat-specific genes, representing processes such as water homeostasis, cytokine-mediated signaling, and glycerolipid metabolism. Similarly, for humans, we observed five communities enriched for human-specific genes, including processes such as biosynthesis of carbohydrate derivatives and DNA damage response. Within the human- and rat-specific communities, only the human-specific cytoplasmic translation branch contained a significant number of genes associated with body-size phenotypes in mice (Figure S6C). The fact that this branch had overlapping functions with the conserved network (chromosome organization) indicated that, while cytoplasmic translation may affect body size across species, it may be more strongly associated with human BMI.

## DISCUSSION

Understanding the shared etiology of a trait across species is crucial for leveraging the many experimental advantages of animal models. Rodent models have long been used to clarify monogenic and polygenic regulation of BMI and obesity.^60–65^ Likewise, outbred rodent populations are also being used for genome-wide studies of complex traits such as BMI,^11,16,37,66,67^ building on successes in human populations. However, similar to the challenge of translating GWAS results between different human populations,^68^ cross-species translation is confounded by numerous factors, both logistical and genetic. Logistical factors include the confounding impact of the environment on human genetic studies and the opaque alignment between model organism paradigms and human traits. Genetic limitations include the incomplete power of GWAS, the potential lack of common variation in genes important for the trait under study, differences in genomic architecture, difficulty in determining the causal gene from loci implicated by GWASs, and imperfect knowledge of gene orthology between species. All these factors limit the translation of genetic findings across species.

Here we have demonstrated that network biology provides a foundation for addressing these limitations, clarifying the extent to which genetic findings in rodent species recapitulate those of human genetic studies. Using a network co-localization framework (NetColoc), we showed that variants affecting different genes in the human and rat populations converge on a conserved BMI network (Figures 2B, 3A, and S3). In doing so, we successfully extended this approach beyond the original application (to compare two human traits) to analyze a putatively similar trait in two different species.^34^ In addition to connecting the disparate gene sets from humans and rats, our network approach expands the set of implicated genes in each species; these network-implicated genes validate well in mouse genetic studies and are critical for understanding functional roles within the conserved BMI network.

The conserved BMI network shows that BMI shares common etiology between rats and humans and provides an opportunity to explore the similarities and differences in BMI regulation in the two species. The structure of the conserved BMI network reveals distinct communities of genes that correspond to distinct underlying mechanisms of BMI common to humans and rats, with particular emphasis on nervous system processes (Figures 3A, 3B, and 4). Many of the systems are corroborated by known mechanisms of BMI in humans or rodents, but the conservation of such systems between species was not obvious from the independent GWAS results. For example, the regulation of hormones such as leptin and insulin is a well-studied mechanism underlying obesity due to its role in metabolism and hunger signaling.^69,70^ While human and rat BMI GWAS identified genes encoding different hormone receptors and signaling proteins, the proximity of these genes in the molecular interaction network identifies the conserved role of neuronal hormone signaling. The well-studied monogenic obesity genes *BNDF, MC4R*, and *POMC* were only identified by human GWAS, but such genes are likely important BMI factors in both species; for example, knockout mutations in *Mc4r* produce obesity phenotypes in rats.^71^

The BMI systems map also highlights key hierarchical relationships among functions; for example, gene communities involved in hormone regulation (GSR), regulation of phosphatase activity (RPA), and muscle development and signaling (MSC) all fall within the broader community of synaptic signaling (SS) (Figure 3A). Genes in this community strongly mediate nervous system physiology in mice, particularly via the mediation of protein-protein interactions at the synapse,^72,73^ and synaptic signaling has also been highlighted in rat and human adiposity regulation.^66^ Together with the brain-specific expression of many genes within the conserved BMI network (Figure 6C), these results indicate a central role for nervous system processes in connecting the convergent biology of BMI across species.

It is also crucial to appreciate the differences between species when considering the implications of rodent models in the study of BMI and obesity. While, in some contexts, the conservation of processes such as hormone regulation and muscle development will be sufficient to translate findings, the differences in genes affected by common variants may be consequential for specific applications. It remains to be seen whether species differences in BMI-associated genes are due to differential functional impacts of genetic variants, higher portions of false-positive results, population structure, or a combination thereof.

### Limitations of the study

While the identification of a conserved BMI network from non-overlapping GWAS results from two species demonstrates the utility of a network approach for cross-species translation, there are several limitations of note. First, BMI is an imperfect measure of body composition since it does not differentiate between tissue types or assess metabolic factors. Nevertheless, it is widely used as a normalized body mass metric in humans and rats and therefore provides a convenient, high-powered, polygenic, and heritable trait for cross-species study.

Second, the human and rat populations do not perfectly represent BMI in their respective species. The human population only represents individuals of European ancestry, and the HS rats, while genetically diverse for a model species, are laboratory animals that do not capture all genetic diversity in domestic and wild rats. Third, some of the disparity between the human and rat GWAS results may be attributed to the difference in population size between the human and rat GWASs and the process of mapping loci to genes. A relaxed significance threshold for the rat GWAS helps address the sample size and LD disparity but may introduce a greater proportion of false positives. Further, mapping loci to genes based on genomic proximity may introduce false positives and can identify multiple genes from a single significant locus. In humans, the nearest gene is thought to be causal about 70% of the time^74–76^; however, the more extensive linkage present in the currently available rodent mapping populations may make this nearest-gene approach less accurate. In this respect, we have previously demonstrated that a network approach is robust to up to 80% false positives and boosts signal from potentially noisy inputs.^35^

Finally, our conversion of rat genes to human orthologs and the use of a human interaction network may introduce bias. Although many genes and cellular processes are similar between humans and other mammals, the mapping of orthologous genes is imperfect, and gene and protein interaction patterns can vary between species due to the rewiring of regulatory and protein-protein interaction networks.^33,77,78^ For this reason, work is ongoing to generate independent molecular interaction networks for mammalian species, including mice,^79^ rats,^80^ and cattle.^81^ Network rewiring of interactions also occurs in different tissues and cell types.^77,82,83^ In this study, we used a single global network, meaning we may have missed processes specific to certain contexts.

While future improvements to our methodology and the underlying data will undoubtedly improve our ability to understand cross-species convergence, this work has demonstrated that existing techniques and molecular network knowledge bases already have substantial power to translate GWAS findings from one species to another. We demonstrated that our approach can extend to additional phenotypes beyond BMI and that cross-species network convergence of genetic results is specific to the phenotypes of interest rather than a general property of the method or mammalian genetics as a whole (Figures 2E and S2E). This study can thus act as a road map for future investigations of phenotypes shared across different species. As the quantity, quality, and variety of data generated from model species increase, approaches such as this one have a key role to play in clarifying the translational potential of these various model species across a spectrum of traits related to human health and disease.

## STAR★METHODS

### RESOURCE AVAILABILITY

#### Lead contact

Further information and requests for resources should be directed to the lead contact, Trey Ideker tideker@ucsd.edu.

#### Materials availability

This study did not generate new unique reagents.

#### Data and code availability

- All data analyzed in this paper are publicly available. Accession numbers are listed in the key resources table. Rat genotype and phenotype data have been deposited at the European Variation Archive (Project: EVA: PRJEB63638, Analyses: ENA: ERZ19474633). All networks and systems maps generated in this study are available as a network set on the Network Data Exchange (NDEx: https://doi.org/10.18119/N97G8T). Gene-level GWAS summary statistics are included in Tables S4 and S5.
- All code used for analysis and data visualization is freely available in public repositories. All original code is publicly available at GitHub: https://github.com/sarah-n-wright/CrossSpeciesBMI as of the date of publication and has been deposited at Zenodo (Zenodo: https://doi.org/10.5281/zenodo.7868889).
- Any additional information required to reanalyze the data reported in this paper is available from the lead contact upon request.

### EXPERIMENTAL MODEL AND STUDY PARTICIPANT DETAILS

The N/NIH heterogeneous stock (HS) rats colony was initiated by the NIH in 1984 using the following eight inbred founder strains: ACI/N, BN/SsN, BUF/N, F344/N, M520/N, MR/N, WKY/N, and WN/N,^101^ which have previously been shown to differ significantly for adiposity traits.^11^ Rats were housed in micro-isolation cages in a conventional facility using autoclaved bedding (sani-chips from PJ Murphy). They were provided reverse osmosis water chlorinated to 2–3 ppm, and had *ad libitum* access to diets as listed below.

The rats used for this study are part of a large multi-site project focused on genetic analysis of behavioral phenotypes related to drug abuse (www.ratgenes.org), and are a larger updated cohort from previously published.^37^ HS rats bred at NMcwi:HS colony (RRID:RGD_2314009) (Medical College of Wisconsin (MCW), WI) were sent to three institutions throughout the United States: University of Tennessee Health Science Center (TN), University at Buffalo (NY), and University of Michigan (MI). The NMcwi:HS breeding colony consisted of 64–96 breeding pairs, bred from 8 founder inbred strains (the number of breeding pairs varied over the course of the experiment). No more than 2 siblings were sent to the same Project, and these siblings had different sex. Rats were shipped at 3–6 weeks of age, and each site received multiple shipments over five years (10/27/2014–03/07/2019). In these datasets, the information was aggregated from multiple projects across different centers, leading to four cohorts, with siblings present in different phenotyping centers. Rats in TN were fed Teklad Irradiated LM-485 Mouse/Rat Diet; rats in NY were fed Envigo Teklad 18% Protein Rodent Diet, and rats in MI were fed Labdiet Picolab Laboratory Rodent Diet Irradiated.

Rats were exposed to a different battery of behavioral testing at each site (Table S3), followed by euthanasia, which occurred at different ages at each site. All phenotypes presented in this paper were collected at the time of euthanasia, in roughly equal numbers of male and female rats. Briefly, in MI, rats were housed in trios, exposed to a single modest dose of cocaine (15 mg/kg) each day for five days, and then euthanized, weighed, and body length was measured 4–7 days after the final cocaine exposure (89 s.d. 6 days of age). In NY, rats were housed in pairs, tested for multiple behaviors over 16 weeks, exposed to a modest dose of cocaine (10 mg/kg) once daily for three days, and then euthanized, weighed, and body length was measured 7–10 days after the last dose of cocaine (198 s.d. 13 days of age). In TN, there were two separate cohorts: breeders (sent from MCW) and experimental rats (bred in TN). Female breeders had mostly one, sometimes two litters and underwent no behavioral testing. After breeding was completed, males and females were euthanized, weighed, and body length was measured (169 s.d. 34 days of age). The experimental rats were tested for multiple behaviors, exposed to nicotine (self-administration, resulting in a range of doses) for 12 days, and euthanized, weighed, and body length was measured 10 days after the final dose of nicotine (73 s.d. 12 days of age). All protocols were approved by the Institutional Animal Care and Use Committees (IACUC) for each of the relevant institutions.

### METHOD DETAILS

#### HS rat phenotyping

Several days after the completion of behavioral experiments (males and females), rats were fasted overnight (17 ± 2 h), and body weight was measured. Under anesthesia (pentobarbital at MI and NY, isoflurane at TN), the rats were measured for body length (from nose to base of the tail (tail length not included in the measurement of the body length)), which we then used to calculate body mass index. BMI was calculated as: (body weight/body length^2^) × 10. Several tissues were dissected and weighed, including spleens, which were used for DNA extraction. All protocols were approved by the Institutional Animal Care and Use Committees (IACUC) for each of the relevant institutions.

#### HS rat DNA sequencing

HS rat DNA was extracted from spleen tissue using the Agencourt DNAdvance Kit (Beckman Coulter Life Sciences, Indianapolis, IN). All genomic DNA quality and purity was assessed by NanoDrop 8000 (Thermo Fisher Scientific, Waltham, MA). Sample DNA, PstI barcoded adapters, and NlaIII Y-adapters were combined in a 96-well plate and allowed to evaporate at 37°C overnight. The PstI adapter barcode is “in-line,” such that each sequencing read from a given sample contains both the PstI overhang sequence (4bps) and a unique adapter sequence (4–8bps) prior to the beginning of the insert sequence. Sample DNA and adapters were reeluted on day two with a PstI/NlaIII digestion mix and incubated at 37°C for 2 h to allow for complete digestion. Ligation reagents were then added and incubated at 16°C for 1 h to anneal the adapters to the DNA fragments, followed by a 30-min incubation at 80°C to inactivate the enzymes. Sample libraries were purified using a plate from a MinElute 96 UF PCR Purification Kit (QIAGEN Inc., Hilden, Germany), vacuum manifold, and ddH2O. Once re-eluted, libraries were quantified in duplicate with Quant-IT PicoGreen (Thermo Fisher Scientific, Waltham, MA) and pooled to the desired level of multiplexing (i.e., 12, 24, or 48 samples per library). Each pooled library was then concentrated by splitting the pooled volume across 2–3 wells of the MinElute vacuum plate and resuspending the library at desired volume for use in the Pippin Prep. The concentrated pool was quantified to ensure the gel cassette was not overloaded with DNA (0.5mg). The pool was then loaded into the Pippin Prep for size selection (300–450 bps) using a 2% agarose gel cassette on a Pippin Prep (Sage Science, Beverly, MA). Size-selected libraries were then PCR amplified for 12 cycles to add the Illumina sequencing primers and increase the quantity of DNA, concentrated again, and checked for quality on an Agilent 2100 Bioanalyzer with a DNA 1000 Series II chip (Agilent Technologies, Santa Clara, CA). Bioanalyzer results were used to ensure sufficient library concentration and to identify excessive primer dimer peaks. Each library was run on a single flow cell lane on an Illumina HiSeq 4000 with 100bp single-end reads at the Institute for Genomic Medicine (IGM) Genomics Center (University of California San Diego, La Jolla, CA).

#### Data acquisition

The summary statistics for human body mass index and height were obtained from the GIANT Consortium (GIANT consortium data files), computed via a meta-analysis of GWAS results representing an average of 681,275 (BMI) and 693,529 (height) individuals of European ancestry.^38^ Summary statistics for all human negative control traits were obtained from the Neale Lab Round 2 UK Biobank (UKB) GWAS^42^ (http://www.nealelab.is/uk-biobank). Phenotype codes are as follows: FEV1: forced expiratory volume per second (20153_irnt), AR: allergic rhinitis symptoms (6152_9), SMOK: smoking status - never smoked cigarettes (20116_0), Neo: non-cancer neoplasms (C3_OTHER_SKIN). Negative control traits were selected to have a substantial number of implicated human genes (n>150), a similar SNP heritability to BMI $\left(0.15<h^{2}<0.35\right)$, and minimal genetic correlation with BMI $\left(\left|r_{g}\right|<0.1\right)$. These estimates were obtained from the UKB Heritability browser (https://nealelab.github.io/UKBB_ldsc/h2_browser.html) and UKB Genetic Correlation browser (https://ukbb-rg.hail.is/), both generated by the Neale Lab.^42^ Bulk human-rat ortholog mapping from HGNC Comparison of Orthology Predictions (HCOP)^102,103^ was downloaded from the European Bioinformatics Institute (http://ftp.ebi.ac.uk/pub/databases/genenames/hcop/) on June 26, 2022. Mouse genotype-phenotype associations and the Mammalian Phenotype Ontology were downloaded from the Mouse Genome Database^44^ (http://www.informatics.jax.org/) on April 24, 2022. Datasets acquired: Phenotype-Genotype Associations (MGI_PhenoGenoMP.rpt); Marker Information (MRK_List2.rpt); Human Ortholog Mapping (MRK_List2.rpt); Mammalian Phenotype Ontology^84^ (MPheno_OBO.ontology). Specific mappings between conserved BMI network genes and BMI-relevant phenotypes in MGD can be found in Table S1. Stable versions of Mouse Genome Database reference data used in this study are contained in the code repository (Zenodo: https://doi.org/10.5281/zenodo.7868889).

#### Molecular interaction networks

The Parsimonious Composite Network^40^ (PCNet v1.3) was obtained from the network data exchange (ndexbio.org), NDEx: 4de852d9–9908-11e9-bcaf-0ac135e8bacf. PCNet is a molecular interaction resource formed from integrating 21 interaction databases that contain various evidence types, including physical protein-protein, genetic, co-expression, and co-citation evidence. Each interaction in PCNet is supported by at least two of the component databases, a threshold chosen to maximize the ability of PCNet to perform gene set recovery tasks via network propagation. All human genes and human orthologs of rat genes were mapped to the nodes of PCNet via gene symbols. Rat interactome data was downloaded from STRING^43^ (v11.5, https://string-db.org/cgi/download.pl) and filtered to only interactions in *R. norvegicus*. We defined high-confidence interactions as those with a combined score >700 and mapped all network node identifiers to rat gene symbols using STRING’s protein information (10116.protein.info. v11.5.txt.gz). All rat genes and rat orthologs of human genes were mapped to the nodes of the high-confidence rat interactome via rat symbols.

### QUANTIFICATION AND STATISTICAL ANALYSIS

#### HS rat genotyping, variant calling & imputation

The PstI adapter barcodes were used to demultiplex FASTQ files into individual sample files. Demultiplexing was completed using FASTX Barcode Splitter v0.0.13 (http://hannonlab.cshl.edu/fastx_toolkit/, RRID: SCR_005534). Reads were discarded if they could not be matched with any barcode (maximum of 1 mismatch allowed) or they lacked the appropriate enzyme cut site. Samples with less than two million reads after demultiplexing were discarded. Read quality was assessed using FastQC v0.11.6.^85^ Reads were trimmed of the barcode, adapter sequences, and low-quality base pairs at the ends of reads using FASTX Clipper/Trimmer/Quality Trimmer tools v0.0.13 (http://hannonlab.cshl.edu/fastx_toolkit/, RRID: SCR_005534). A base quality threshold of 20 was used and reads shorter than 25bp were discarded.

The *R. norvegicus* genome assembly rn6 was used as the reference genome for read alignment with the Burrows-Wheeler Aligner v0.7.5a (BWA)^86^ using the mem algorithm. We then used GATK IndelRealginer v3.5^87^ to improve alignment quality by locally realigning reads around a reference set of known indels in 42 whole-genome sequenced inbred rat strains, including the eight HS progenitor strains.^104^

Variants were called, and genotype likelihoods were computed at variant sites using ANGSD v0.911, under the SAMtools model for genotype likelihoods (ANGSD-SAMtools).^88,89^ Further, using ANGSD-SAMtools, we inferred the major and minor alleles (-domajorminor 1) from the genotype likelihoods, retaining only high confidence polymorphic sites (-snp_pval 1e-6), and estimated the allele frequencies based on the inferred alleles (-domaf 1). We discarded sites missing read data in more than 4% of samples (–minInd). Additionally, we tested multiple thresholds for minimum base (-minQ) and mapping (-minMapQ) qualities. Variants for X- and Y chromosomes were not called. Prior to GWAS, SNPs in high linkage disequilibrium were removed using PLINK v1.9^90,91^ with an r^2^ cutoff of 0.95.

Beagle v4.1^92^ was used to improve the genotyping within the samples without the use of an external reference panel. Missing and low-quality genotypes were imputed by borrowing information from other individuals in the dataset with high-quality information at these same variant sites. To verify the quality of the “internally” imputed genotypes prior to imputing SNPs from the 42 inbred strain reference panel^104^ we checked concordance rates for the 96 HS animals with array genotypes, examined the TSTV ratio, and assessed whether the sex as recorded in the pedigree records agreed with the sex empirically determined by the proportion of reads on the X chromosome out of the total number of reads. We also identified Mendelian errors using the –mendel option in PLINK and known pedigree information for 1,136 trios from 214 families within the HS sample. Using the fraction of the trios that were informative for a given SNP and the equation 1−(1−2p(1−p))3, where p represents the minor allele frequency of the allele, we formed curves for the distributions of the expected number of Mendelian errors for both SNPs and samples and chose the inflection points as thresholds for the number of Mendelian errors allowed.

Only variants previously identified in the eight HS founder strains were retained as we expected the polymorphisms in our samples to be limited to the variation present in the founders.^104,105^ Estimated haplotypes from our dataset for imputation with multiple different reference panels were calculated previously,^106^ and were applied to this dataset.

For imputation, we used a reference panel calculated previously^106^ from a combination of existing sequencing and array genotyping data from the HS rat founder and other inbred laboratory rat strains.^104^ Genotype data underwent QC and were phased by Beagle into single chromosome haplotype files. Imputation by IMPUTE2 was performed in 5Mb windows using the aforementioned reference panels and genetic maps. Final genotypes for all samples are reported in EVA: PRJEB63638.

#### HS rat genome-wide association

Each trait within each research site was quantile-normalized separately for males and females; an approach similar to using sex as a covariate. Other relevant covariates (including age, batch number, and dissector) were identified for each trait, and covariate effects were regressed out if they were significant and if they explained more than 2% of the variance (BMI covariates: age and test center for all rats; additionally for TN, experimental or breeders, and TN experimental: behavioral experiment, Technician JS, and for TN breeder: Technician XH. Body length covariates: age, test center for all rats; additionally for TN experimental: Technician JS, TN experiment, and for TN breeders: age, Technician XH). Residuals were then quantile-normalized again, after which the data for each sex and site were pooled prior to further analysis. This approach removed mean differences due to sex; however, it did not attempt to model gene-by-sex interactions. By quantile-normalizing the three centers separately, we also addressed the numerous and confounded differences among the three cohorts, such that quantitative trait loci (QTLs) identified in the current study are resistant to environmental influences that differed among the sites. Raw phenotypes and post-regression quantile-normalized values for body length without tail and body mass index without tail are reported in EVA: PRJEB63638.

The software GCTA^94^ was used to perform the GWAS analysis. First, we pre-calculated a genetic relatedness matrix (GRM) from autosomal chromosomes (–autosome –make-grm-bin). We then performed the GWAS with a linear mixed model (–mlma), using the GRM to account for complex family relationships within the HS rat population (–mlma-subtract-grm). This method employs a Leave One Chromosome Out (LOCO) method to avoid proximal contamination.^107,108^

#### Rat SNP-to-gene mapping

We assigned gene-level significance values by taking the lowest p value of any GWAS variant within a ± 10kb region encompassing each gene region. For each gene’s set of assigned SNPs, the SNP with the lowest p value was assigned as the lead SNP for that gene and used to assess the significance of the gene (Table S4). Using a threshold of p < 1×10^−4^, we defined sets of rat seed genes significantly associated with rat body mass index (BMI) and rat body length (without tail; BL). For BMI, 20,079 top SNPs were assigned to 23,884 rat genes, with 3,108 being assigned to more than one gene. After significance filtering, 413 lead SNPs were assigned to 476 genes, with 54 SNPs being assigned to more than one of the 476 significant genes identified. Rat genes were then mapped to human orthologs using the HCOP^102,103^ search tool (https://www.genenames.org/tools/hcop/), which incorporates orthology information from 16 databases. We required that the ortholog be present in at least three databases to map a gene. To plot the position of rat seed genes in the rat genome (Figure S1), each gene’s genomic range ±10kb was plotted in a single column per chromosome, and manually aligned to each chromosome. Due to variable genomic ranges listed for some genes, the smallest start site and greatest end site from those listed in Table S4 were used to define the genomic range.

#### Human SNP-to-gene mapping

We generated gene-level significance values (Table S5) from the SNP-level summary statistics for each human trait using the PASCAL algorithm^95^ using default parameters. This approach assigns SNPs to a gene if they fall within a window of ±50kb of the gene region and then calculates an aggregate p value after correcting for linkage disequilibrium. To model the expected distribution of SNP p values, an SNP-by-SNP correlation matrix is formed from the 1,000 Genomes Project^109^ reference data for European individuals (1KG-EUR) for all *n* SNPs in the gene region and used as the basis of a multivariate normal null distribution, allowing calculation of z-scores for each SNP. A weighted sum of $\chi_{1}^{2}$-distributed random variables is used to model the sum of all z-scores within a gene region, with the resulting gene p value of the sum of chi-squared statistics calculated using the Davies algorithm (or Farebrother algorithm) for weighted sums of independent $\chi_{1}^{2}$-distributed random variables.^110,111^ Following a Bonferroni correction of the PASCAL p values, we defined sets of trait-associated genes using a threshold of q < 0.05.

#### Network propagation and co-localization

We used the Python package NetColoc^34,35^ (https://pypi.org/project/netcoloc/) for network propagation and co-localization. The sets of significant trait-associated genes from GWAS were used as “seed” genes for network propagation using a Random Walk with Restart^41^ algorithm. This algorithm models the diffusion of heat along the edges of a network from an initial set of “hot” seed genes. In each iteration, a unit of heat is added to each seed gene, and the heat diffuses from each gene to its neighbors. To conserve the total heat within the system, a constant fraction of heat dissipates from each gene with each iteration. The heat diffusion process converges to a closed-form solution^35^ described by Equation 1:

(Equation 1) $$F=(I-\alpha W{)}^{-1}(1-\alpha )Y_{0}$$

Where F is the stable heat vector for all nodes, $Y_{0}$ is the vector of seed genes, W is the column-normalized adjacency matrix of the network (PCNet), and α∈(0,1) is the dissipation constant. Following network propagation with α=0.5, we calculated a network proximity score (NPS) for each gene in the network by comparing the observed results to a null distribution. The null distribution was formed by propagating 1,000 randomly selected seed gene sets. Each set was sampled to preserve the size and degree distribution of the original input set. As previously implemented,^112^ we binned all genes in the network by degree with a minimum of 10 nodes per bin. For each gene g, the NPS was calculated as a Z score comparing the observed heat at that gene $F_{g,S}$ after network propagation of gene set S, to the mean of the null distribution heats at that gene ${\vec{F}}_{g,rand}$ as given by Equation 2:

(Equation 2) $$NPS_{g,S}=\frac{log\left(F_{g,S}\right)\;-\;\langle \log\;\left({\vec{F}}_{g,rand}\right)\rangle }{\sigma \left(\log\;\left({\vec{F}}_{g,rand}\right)\right)}$$

Where 〈〉 denotes the mean of a vector, and σ denotes the standard deviation of a vector. All heat values are log-transformed to ensure the distributions are approximately normal.

NetColoc recommends fewer than 500 input seed genes given the sample space of PCNet (~18,000 genes). Therefore, we employed a weighted sampling procedure for any trait having more than 500 significantly associated genes. We sampled 500 genes from the set of all significant genes (weighted by −log_10_p from GWAS) and ran the propagation analysis from this subset. After 100 repetitions, the 75% percentile NPS score was selected to approximate a consensus score for each gene.

From input seed genes for humans and rats, we independently calculated $NPS_{h}$ (human) and $NPS_{r}$ (rat) for each trait. We then defined a gene as colocalized between two species if it had high proximity to inputs in both species. Therefore, we defined the combined network proximity $NPS_{hr}$ as the product of the independent species scores in Equation 3:

(Equation 3) $$NPS_{hr}=NPS_{h}\circ NPS_{r}$$

We repeated this approach using our high-confidence rat interaction network from STRING^43^ (v11.5). All human input seed genes were mapped to rat gene symbols using the bulk download human-rat ortholog mapping from HCOP.^102,103^ Where multiple complete mappings existed for a gene, the one supported by the most databases was selected.

#### Definition of the conserved BMI network

From $NPS_{hr}$_,_ we selected genes with high proximity scores in both species to define the conserved cross-species network using thresholds: $NPS_{hr}>3,\;NPS_{h}>1$, and $NPS_{r}>1$ (Figure S5A). To calculate the significance of the network co-localization, we compared the conserved network size and the mean $NPS_{hr}$ to a permuted null distribution. We permuted the labels of $NPS_{r}$ 10,000 times, and each time calculated the mean $NPS_{hr}$ across all genes and the number of genes passing the above thresholds. For genes present in both the human and rat input sets, labels were permuted separately to maintain the higher expected distribution for these genes. The significance of the conserved network size and mean $NPS_{hr}$ was calculated by Z-test.

#### Definition of the species-specific BMI networks

We also defined human-specific (n=925 genes) and rat-specific (n=688 genes) BMI networks to investigate the biology underlying BMI in only one species (Figures S5B and S5C). We used modified individual and combined NPS thresholds to capture genes with high NPS in only one species. For the individual thresholds on $NPS_{h}$ and $NPS_{r}$, we set cutoffs based on the NPS distributions, calculated from non-seed genes. For the same-species threshold (e.g., the threshold on $NPS_{r}$ for defining the rat-specific network), we required that the NPS be a minimum of one standard deviation above the mean in that species. Therefore, for the human-specific network, we required $NPS_{h}>1.5$, and for the rat-specific network, we required $NPS_{r}>1$. For the other-species threshold (e.g., the threshold on $NPS_{h}$ for defining the rat-specific network), we required that the NPS be below the mean of all scores in that species, resulting in a threshold of $NPS_{r}<0$ for the human-specific network and $NPS_{h}<0.5$ for the rat-specific network. We further defined combined thresholds as $NPS_{r}(NPS_{h}-1)<\;-2$ for the rat-specific network and $NPS_{h}(NPS_{r}-1)<\;-4$ for the human-specific network. Compared to the $NPS_{hr}>3$ threshold for the conserved network, the shift in directionality captured genes with high NPS in one species but not the other, and the NPS−1 transformation allowed the inclusion of genes with slightly positive, but below the mean, NPS in the other species. We set the combined thresholds (−2 and −4) to generate subnetworks of comparable size (500–1000 genes) to the conserved network, resulting in a stricter threshold for the human-specific threshold (Figure S5D). Finally, we defined an expanded network comprising all genes with an NPS score one standard deviation above the mean in at least one species ($NPS_{h}>1.5$ or $NPS_{r}>1$) to enable the analysis of the conserved and species-specific networks under a common framework (n=6025). By design, this expanded network contained the conserved, rat-specific, and human-specific networks as subnetworks.

#### Gene set enrichment analysis

Sets of genes were assessed for biological function by quantifying significant enrichment in Gene Ontology (GO) Biological Process terms using gProfiler.^97^ The gProfiler g:GOSt method maps genes to functional terms and assesses the statistical significance of functional enrichment. We maintained communities with 1) at least five genes, 2) at least three genes mapped to a GO term of size 50–1,000 genes, and 3) an FDR-corrected gene-set enrichment p value <1×10^−3^. Enrichment results for seed genes and network sets are included in Table S2.

#### Construction of multiscale systems maps

We generated a multiscale systems map of the conserved BMI network using the Hierarchical community Decoding Framework (HiDeF)^96^ algorithm as implemented in Cytoscape’s Python package CDAPS.^113^ HiDeF is an efficient hierarchical community detection method that makes use of persistent homology to identify structures at all scales simultaneously. First, the interaction network is reformulated as a fully connected similarity network and scanned across a range of modularity resolutions to identify densely connected network communities using a Louvain algorithm for community detection. At high resolutions, community detection tends to identify a larger number of small communities, whereas at low resolutions it tends to identify a smaller number of large communities. From this pool of all candidate communities, persistent communities are then defined as those that are redundantly identified across resolutions based on pairwise Jaccard similarities. Finally, the communities are organized into a hierarchical structure by finding those that are partially or fully nested within others based on the *containment index* (CI, Equation 4) which measures the overlap between communities v and w.

(Equation 4) $$CI(v,w)=\frac{\left|s(v)\;\cap s\;(w)\right|}{\left|s(w)\right|}$$

If CI(v,w) is greater than a threshold σ, then an edge is added in the hierarchy from v to w. Any redundant relations are then reduced to produce the final multiscale hierarchy. To identify the conserved BMI systems map, we implemented HiDeF with maximum resolution set to 10, and all other parameters set to defaults. The resulting communities were annotated based on significantly enriched GO Biological Processes using gProfiler,^97^ followed by manual curation (See gene set enrichment analysis). For communities containing multiple seed genes colocated on the same chromosome arm within 250kb, we verified that these seed genes were drawn from multiple independent loci (Table S6) to ensure that systems did not over-represent a single locus.

We performed the same hierarchical community detection and annotation procedure for the expanded BMI network, retaining all communities with more than ten genes to form the expanded BMI Systems Map. For each community, we calculated the enrichment of genes from each of the conserved, rat-specific, and human-specific networks via a hypergeometric test followed by Benjamini-Hochberg correction of p values.

#### Mouse variant validation

We accessed mouse knockout, knock-in, and mutation data from the Mouse Genome Database,^114^ including genotype-phenotype associations mapped to the Mammalian Phenotype Ontology^84^ (MPO), similar to the analysis previously published.^34,35^ We extracted mouse genes from the listed alleles, including the target genes from conditional genotypes, and mapped them to human orthologs using MGD’s marker and ortholog mappings. We used a set of BMI-relevant phenotypes within the MPO (MP:0001697 - abnormal embryo size; MP:0010866 - abnormal prenatal body size; MP:0003956 - abnormal postnatal body size; MP:0005451 - abnormal body composition) to assess relevance to BMI. For the full conserved network and each community, we reported the odds ratio of the number of genes linked to a phenotype in the MGD and tested this value for enrichment using a Fisher’s Exact Test. The validation rate of genes within a network was defined as the number of genes associated with at least one BMI-relevant phenotype divided by the number of genes with at least one normal or abnormal phenotype reported in MGD. The MPO is hierarchical, so we considered all genes associated with a phenotype to be associated with all parent phenotypes in the MPO hierarchy. When performing multiple simultaneous tests, we used the Benjamin-Hochberg (BH) correction to calculate adjusted q values with FDR = 0.05.

We tested all communities in the conserved and expanded systems maps for the enrichment of genes with mouse orthologs associated with BMI-relevant phenotypes in MGD. For the conserved systems map, we chose communities with the highest significant odds ratios (p<0.05) for at least one BMI-relevant phenotype for further investigation. To assess the functional effects of the gene communities, for each phenotype term, we calculated the positive predictive value (PPV) from the set of genes in the community (community), and the set of genes associated with the phenotype in MGD (phenotype) as:

(Equation 5) $$PPV_{phenotype}=\frac{|community\;\cap \;phenotype|}{|phenotype|}$$

We took a top-down approach from the root “mammalian phenotype” term (MP:0000001). At each level of the hierarchy, we kept all child terms with $PPV_{child}>PPV_{parent}$ and performed a Benjamini-Hochberg correction on the enrichment p values for the set of child terms for each parent phenotype. A representative subset of higher-level terms was selected to enable readability in the phenotype enrichment heatmaps (Figures 4 and 5D).

#### Tissue enrichment

To assess the tissue-specific expression of genes in the conserved and species-specific networks, we used the Tissue-Specific Enrichment Tool (TSEA,^98^ http://genetics.wustl.edu/jdlab/tsea/) and the TissueEnrich^99^ tool (http://tissueenrich.gdcb.iastate.edu/). We used TSEA to calculate the enrichment of gene sets for selective expression in 25 tissue types based on human GTEx^115^ data, and defined an FDR-corrected p value threshold of q < 0.05 as a significant enrichment. We used TissueEnrich to calculate the enrichment of mouse orthologs of gene sets for tissue-specific genes in 17 mouse tissue types based on mouse ENCODE data.^116^ We defined significant enrichments as those with an FDR-corrected p value threshold of q < 0.05.

#### Network visualization

We generated all hierarchy and network figures using Cytoscape.^100^ Tree and radial layouts were used as the basis for all hierarchy figures (Figures 3A, 5B, 6E, S3B, and S6B), and a spring-embedded layout algorithm was used to determine the layouts of network and subnetwork figures (Figures 3C, 4A, 5A, S3A, and S4). For communities in systems maps, node graphics were used to display the source of the genes, and node size was used to indicate the number of genes. For gene networks, node color was used to indicate the source of genes.

## Supplementary Material

1

2

3

4

5

6

7

## Figures and Tables

**KEY RESOURCES TABLE:** 

REAGENT or RESOURCE	SOURCE	IDENTIFIER

Deposited data		

GWAS summary statistics (Human BMI, height)	Yengo et al.^38^	https://portals.broadinstitute.org/collaboration/giant/index.php/GIANT_consortium_data_files
Rat genotypes and phenotypes (Rat BMI, body length)	This paper	EVA: PRJEB63638 (Project); ENA: ERZ19474633 (Analysis)
Rat gene-level summary statistics (BMI, body length)	This paper	Table S4
Human gene-level summary statistics (BMI, Height)	This paper	Table S5
Control GWAS summary statistics	Neale Lab^42^	http://www.nealelab.is/uk-biobank; Zenodo: https://doi.org/10.5281/zenodo.7186871
PCNet Interactome (v1.3)	Huang et al.^40^	Ndexbio.org, NDEx: 4de852d9-9908-11e9-bcaf-0ac135e8bacf
STRING High Confidence Rat Interactome	Szklarczyk et al.,^43^ this paper	Ndexbio.org, NDEx: 880c7d8c-f5ad-11ec-ac45-0ac135e8bac
Conserved Human-Rat BMI Network	This paper	Ndexbio.org, NDEx: https://doi.org/10.18119/N9102V
Conserved Human-Rat BMI Systems Map	This paper	Ndexbio.org, NDEx: https://doi.org/10.18119/N9RG73
Human-Specific BMI Network	This paper	Ndexbio.org, NDEx: https://doi.org/10.18119/N9MS56
Rat-Specific BMI Network	This paper	Ndexbio.org, NDEx: https://doi.org/10.18119/N9H03K
Expanded BMI Systems Map	This paper	Ndexbio.org, NDEx: https://doi.org/10.18119/N9C89C
Expanded BMI Network	This paper	Ndexbio.org, NDEx: https://doi.org/10.18119/N9W90Z
Mouse genotype-phenotype associations	Blake et al.^44^	https://https://www.informatics.jax.org/downloads/
Mammalian Phenotype Ontology	Smith and Eppig^84^	https://https://www.informatics.jax.org/downloads/

Experimental models: Organisms/strains		

Heterogeneous Stock (HS) Rats	Medical College of Wisconsin, WI	MCW: NMcwi:HS #2314009; RRID

Software and algorithms		

CrossSpeciesBMI v.1.0.0	This paper	Zenodo: https://doi.org/10.5281/zenodo.7868889
FASTX-Toolkit v0.0.13	Hannon Lab, 2010	RRID: SCR_005534; http://hannonlab.cshl.edu/fastx_toolkit/
FastQC v0.11.6	Babraham Bioinformatics^85^	RRID: SCR_014583; http://www.bioinformatics.babraham.ac.uk/projects/fastqc/
Burrows-Wheeler Aligner (BWA) v0.7.5a	Li and Durbin^86^	RRID: SCR_010910; http://bio-bwa.sourceforge.net/
GATK v3.5	McKenna et al.^87^	RRID: SCR001876; https://software.broadinstitute.org/gatk/
ANGSD-SAMtools	Korneliussen et al.^88^ & Durvasula et al.^89^	RRID: SCR_021865; https://github.com/ANGSD/angsd;
PLINK v1.9	Purcell et al.^90^ & Chang et al.^91^	RRID: SCR_001757; https://www.cog-genomics.org/plink/
BEAGLE v4.1	Browning and Browning^92^	RRID: SCR_001789; http://faculty.washington.edu/browning/beagle/beagle.html
IMPUTE2	Howie et al.^93^	RRID: SCR_013055; https://mathgen.stats.ox.ac.uk/impute/impute_v2.html
GCTA	Yang et al.^94^	https://yanglab.westlake.edu.cn/software/gcta/#Overview
PASCAL	Lamparter et al.^95^	https://www2.unil.ch/cbg/index.php?title=Pascal
Python 3.9.13	N/A	https://www.python.org/downloads/release/python-3913/
NetworkX 2.8.4	N/A	RRID: SCR_016864; https://networkx.org/
NetColoc v0.1.6.post1	Rosenthal et al.^34^	https://pypi.org/project/netcoloc/0.1.6.post1/
HiDeF v1.0.0	Zheng et al.^96^	https://apps.cytoscape.org/apps/cycommunitydetection
Gprofiler	Raudvere et al.^97^	RRID: SCR_006809; https://biit.cs.ut.ee/gprofiler/
TSEA	Dougherty et al.^98^	http://genetics.wustl.edu/jdlab/tsea/
TissueEnrich	Jain and Tuteja^99^	http://tissueenrich.gdcb.iastate.edu/
Cytoscape 3.9.1	Shannon et al.^100^	RRID: SCR_003032; https://cytoscape.org

## Highlights

- Disparate genetics in humans and rats converge on a conserved molecular network
- A systems map of 27 interacting gene communities defines shared BMI biology
- Conserved BMI genes are linked to mechanisms of BMI via the Mouse Genome Database
- Neuronal pathways are a critical shared mechanism of BMI in humans and rodents

## References

[1] Mestas J, and Hughes CCW (2004). Of mice and not men: differences between mouse and human immunology. J. Immunol. 172, 2731–2738. 14978070 10.4049/jimmunol.172.5.2731

[2] Perlman RL (2016). Mouse models of human disease: An evolutionary perspective. Evol. Med. Public Health 2016, 170–176. 27121451 10.1093/emph/eow014 PMC4875775

[3] Even PC, Virtue S, Morton NM, Fromentin G, and Semple RK (2017). Editorial: Are Rodent Models Fit for Investigation of Human Obesity and Related Diseases? Front. Nutr. 4, 58. 29250524 10.3389/fnut.2017.00058 PMC5717807

[4] Tam V, Patel N, Turcotte M, Bossé Y, Paré G, and Meyre D (2019). Benefits and limitations of genome-wide association studies. Nat. Rev. Genet. 20, 467–484. 31068683 10.1038/s41576-019-0127-1

[5] French JD, and Edwards SL (2020). The Role of Noncoding Variants in Heritable Disease. Trends Genet. 36, 880–891. 32741549 10.1016/j.tig.2020.07.004

[6] Reynolds T, Johnson EC, Huggett SB, Bubier JA, Palmer RHC, Agrawal A, Baker EJ, and Chesler EJ (2021). Interpretation of psychiatric genome-wide association studies with multispecies heterogeneous functional genomic data integration. Neuropsychopharmacology 46, 86–97. 32791514 10.1038/s41386-020-00795-5 PMC7688940

[7] Mignogna KM, Bacanu SA, Riley BP, Wolen AR, and Miles MF (2019). Cross-species alcohol dependence-associated gene networks: Co-analysis of mouse brain gene expression and human genome-wide association data. PLoS One 14, e0202063. 31017905 10.1371/journal.pone.0202063 PMC6481773

[8] Li Z, Votava JA, Zajac GJM, Nguyen JN, Leyva Jaimes FB, Ly SM, Brinkman JA, De Giorgi M, Kaul S, Green CL, (2020). Integrating Mouse and Human Genetic Data to Move beyond GWAS and Identify Causal Genes in Cholesterol Metabolism. Cell Metabol. 31, 741–754.e5. 10.1016/j.cmet.2020.02.015 PMC7184639 32197071

[9] Gileta AF, Fitzpatrick CJ, Chitre AS, St Pierre CL, Joyce EV, Maguire RJ, McLeod AM, Gonzales NM, Williams AE, Morrow JD, (2022). Genetic characterization of outbred Sprague Dawley rats and utility for genome-wide association studies. PLoS Genet. 18, e1010234. 35639796 10.1371/journal.pgen.1010234 PMC9187121

[10] Gunturkun MH, Wang T, Chitre AS, Garcia Martinez A, Holl K, St Pierre C, Bimschleger H, Gao J, Cheng R, Polesskaya O, (2022). Genome-Wide Association Study on Three Behaviors Tested in an Open Field in Heterogeneous Stock Rats Identifies Multiple Loci Implicated in Psychiatric Disorders. Front. Psychiatr. 13, 790566. 10.3389/fpsyt.2022.790566 PMC8882588 35237186

[11] Keele GR, Prokop JW, He H, Holl K, Littrell J, Deal A, Francic S, Cui L, Gatti DM, Broman KW, (2018). Genetic Fine-Mapping and Identification of Candidate Genes and Variants for Adiposity Traits in Outbred Rats. Obesity 26, 213–222. 29193816 10.1002/oby.22075 PMC5740008

[12] Rat Genome Sequencing and Mapping Consortium; Baud A, Hermsen R, Guryev V, Stridh P, Graham D, McBride MW, Foroud T, Calderari S, Diez M, (2013). Combined sequence-based and genetic mapping analysis of complex traits in outbred rats. Nat. Genet. 45, 767–775. 23708188 10.1038/ng.2644 PMC3821058

[13] Yang C, Wang Y, Xu W, Liu Z, Zhou S, Zhang M, and Cui D (2019). Genome-wide association study using diversity outcross mice identified candidate genes of pancreatic cancer. Genomics 111, 1882–1888. 30578891 10.1016/j.ygeno.2018.12.011

[14] Long PN, Cook VJ, Majumder A, Barbour AG, and Long AD (2022). The utility of a closed breeding colony of Peromyscus leucopus for dissecting complex traits. Genetics 221, iyac026. 10.1093/genetics/iyac026. 35143664 PMC9071557

[15] Zou J, Gopalakrishnan S, Parker CC, Nicod J, Mott R, Cai N, Lionikas A, Davies RW, Palmer AA, and Flint J (2022). Analysis of independent cohorts of outbred CFW mice reveals novel loci for behavioral and physiological traits and identifies factors determining reproducibility. G3 12, jkab394. 34791208 10.1093/g3journal/jkab394 PMC8728023

[16] Keller MP, Rabaglia ME, Schueler KL, Stapleton DS, Gatti DM, Vincent M, Mitok KA, Wang Z, Ishimura T, Simonett SP, (2019). Gene loci associated with insulin secretion in islets from non-diabetic mice. J. Clin. Invest. 129, 4419–4432. 31343992 10.1172/JCI129143 PMC6763251

[17] Huang W, Campbell T, Carbone MA, Jones WE, Unselt D, Anholt RRH, and Mackay TFC (2020). Context-dependent genetic architecture of Drosophila life span. PLoS Biol. 18, e3000645. 32134916 10.1371/journal.pbio.3000645 PMC7077879

[18] Erickson PA, Weller CA, Song DY, Bangerter AS, Schmidt P, and Bergland AO (2020). Unique genetic signatures of local adaptation over space and time for diapause, an ecologically relevant complex trait, in Drosophila melanogaster. PLoS Genet. 16, e1009110. 33216740 10.1371/journal.pgen.1009110 PMC7717581

[19] Wu KJ, Kumar S, Serrano Negron YL, and Harbison ST (2018). Genotype Influences Day-to-Day Variability in Sleep in Drosophila melanogaster. Sleep 41, zsx205. 29228366 10.1093/sleep/zsx205 PMC6018780

[20] Kos MZ, Carless MA, Blondell L, Leland MM, Knape KD, Göring HHH, and Szabó CÁ (2021). Whole Genome Sequence Data From Captive Baboons Implicate RBFOX1 in Epileptic Seizure Risk. Front. Genet. 12, 714282. 34490042 10.3389/fgene.2021.714282 PMC8417722

[21] Zhang Q, Cai Z, Lhomme M, Sahana G, Lesnik P, Guerin M, Fredholm M, and Karlskov-Mortensen P (2020). Inclusion of endophenotypes in a standard GWAS facilitate a detailed mechanistic understanding of genetic elements that control blood lipid levels. Sci. Rep. 10, 18434. 33116219 10.1038/s41598-020-75612-6 PMC7595098

[22] Letko A, Minor KM, Norton EM, Marinescu VD, Drögemüller M, Ivansson E, Megquier K, Noh HJ, Starkey M, Friedenberg SG, (2021). Genome-Wide Analyses for Osteosarcoma in Leonberger Dogs Reveal the CDKN2A/B Gene Locus as a Major Risk Locus. Genes 12, 1964. 34946912 10.3390/genes12121964 PMC8700858

[23] Hédan B, Cadieu É, Rimbault M, Vaysse A, Dufaure de Citres C, Devauchelle P, Botherel N, Abadie J, Quignon P, Derrien T, and André C (2021). Identification of common predisposing loci to hematopoietic cancers in four dog breeds. PLoS Genet. 17, e1009395. 33793571 10.1371/journal.pgen.1009395 PMC8016107

[24] Washington NL, Haendel MA, Mungall CJ, Ashburner M, Wester-field M, and Lewis SE (2009). Linking human diseases to animal models using ontology-based phenotype annotation. PLoS Biol. 7, e1000247. 19956802 10.1371/journal.pbio.1000247 PMC2774506

[25] Jia P, and Zhao Z (2014). assisted analysis to prioritize GWAS results: principles, methods and perspectives. Hum. Genet. 133, 125–138. 24122152 10.1007/s00439-013-1377-1 PMC3943795

[26] Fong SH, Carlin DE, Ozturk K, Ideker T, Arang N, Bao B, Bennett H, Cai X, Chau K, Fixsen B, (2018). UCSD Network Biology Class & Ideker, T. Strategies for Network GWAS Evaluated Using Classroom Crowd Science. Cell Syst. 8, 275–280. 10.1016/j.cels.2019.03.013 PMC6764759 31022372

[27] Keller MP, Gatti DM, Schueler KL, Rabaglia ME, Stapleton DS, Simecek P, Vincent M, Allen S, Broman AT, Bacher R, (2018). Genetic Drivers of Pancreatic Islet Function. Genetics 209, 335–356. 29567659 10.1534/genetics.118.300864 PMC5937189

[28] Schwartzentruber J, Cooper S, Liu JZ, Barrio-Hernandez I, Bello E, Kumasaka N, Young AMH, Franklin RJM, Johnson T, Estrada K, (2021). Genome-wide meta-analysis, fine-mapping and integrative prioritization implicate new Alzheimer’s disease risk genes. Nat. Genet. 53, 392–402. 33589840 10.1038/s41588-020-00776-w PMC7610386

[29] Carlin DE, Fong SH, Qin Y, Jia T, Huang JK, Bao B, Zhang C, and Ideker T (2019). A Fast and Flexible Framework for Network-Assisted Genomic Association. iScience 16, 155–161. 31174177 10.1016/j.isci.2019.05.025 PMC6554232

[30] Wang Q, Chen R, Cheng F, Wei Q, Ji Y, Yang H, Zhong X, Tao R, Wen Z, Sutcliffe JS, (2019). A Bayesian framework that integrates multi-omics data and gene networks predicts risk genes from schizophrenia GWAS data. Nat. Neurosci. 22, 691–699. 30988527 10.1038/s41593-019-0382-7 PMC6646046

[31] Biran H, Almozlino T, Kupiec M, and Sharan R (2018). A Web Server for Network Propagation. J. Mol. Biol. 430, 2231–2236. 29524510 10.1016/j.jmb.2018.02.025

[32] Bogenpohl JW, Mignogna KM, Smith ML, and Miles MF (2017). Integrative Analysis of Genetic, Genomic, and Phenotypic Data for Ethanol Behaviors: A Network-Based Pipeline for Identifying Mechanisms and Potential Drug Targets. Methods Mol. Biol. 1488, 531–549. 27933543 10.1007/978-1-4939-6427-7_26 PMC5152688

[33] Podder A, Raju A, and Schork NJ (2021). Cross-species and human inter-tissue network analysis of genes implicated in longevity and aging reveal strong support for nutrient sensing. Front. Genet. 12, 719713. 34512728 10.3389/fgene.2021.719713 PMC8430347

[34] Rosenthal SB, Willsey HR, Xu Y, Mei Y, Dea J, Wang S, Curtis C, Sempou E, Khokha MK, Chi NC, (2021). A convergent molecular network underlying autism and congenital heart disease. Cell Syst. 12, 1094–1107.e6. 10.1016/j.cels.2021.07.009. 34411509 PMC8602730

[35] Rosenthal SB, Wright SN, Liu S, Churas C, Chilin-Fuentes D, Chen CH, Fisch KM, Pratt D, Kreisberg JF, and Ideker T (2023). Mapping the common gene networks that underlie related diseases. Nat. Protoc. 18, 1745–1759. 10.1038/s41596-022-00797-1. 36653526 PMC10257754

[36] Kleinert M, Clemmensen C, Hofmann SM, Moore MC, Renner S, Woods SC, Huypens P, Beckers J, de Angelis MH, Schürmann A, (2018). Animal models of obesity and diabetes mellitus. Nat. Rev. Endocrinol. 14, 140–162. 29348476 10.1038/nrendo.2017.161

[37] Chitre AS, Polesskaya O, Holl K, Gao J, Cheng R, Bimschleger H, Garcia Martinez A, George T, Gileta AF, Han W, (2020). Genome-Wide Association Study in 3,173 Outbred Rats Identifies Multiple Loci for Body Weight, Adiposity, and Fasting Glucose. Obesity 28, 1964–1973. 32860487 10.1002/oby.22927 PMC7511439

[38] Yengo L, Sidorenko J, Kemper KE, Zheng Z, Wood AR, Weedon MN, Frayling TM, Hirschhorn J, Yang J, and Visscher PM; GIANT Consortium (2018). Meta-analysis of genome-wide association studies for height and body mass index in ~700000 individuals of European ancestry. Hum. Mol. Genet. 27, 3641–3649. 30124842 10.1093/hmg/ddy271 PMC6488973

[39] Munro D, Wang T, Chitre AS, Polesskaya O, Ehsan N, Gao J, Gusev A, Woods LCS, Saba LM, Chen H, (2022). The regulatory landscape of multiple brain regions in outbred heterogeneous stock rats. Nucleic Acids Res. 50, 10882–10895. 36263809 10.1093/nar/gkac912 PMC9638908

[40] Huang JK, Carlin DE, Yu MK, Zhang W, Kreisberg JF, Tamayo P, and Ideker T (2018). Systematic Evaluation of Molecular Networks for Discovery of Disease Genes. Cell Syst. 6, 484–495.e5. 29605183 10.1016/j.cels.2018.03.001 PMC5920724

[41] Vanunu O, Magger O, Ruppin E, Shlomi T, and Sharan R (2010). Associating genes and protein complexes with disease via network propagation. PLoS Comput. Biol. 6, e1000641. 20090828 10.1371/journal.pcbi.1000641 PMC2797085

[42] Abbott L, Bloom J, Bryant S, Carey C, Churchhouse C, Ganna A, Goldstein J, Howrigan D, King D, Palmer D, (2022). Nealelab/UKBB_ldsc: v2.0.0 (Round 2 GWAS Update). 10.5281/zenodo.7186871.

[43] Szklarczyk D, Gable AL, Nastou KC, Lyon D, Kirsch R, Pyysalo S, Doncheva NT, Legeay M, Fang T, Bork P, (2021). The STRING database in 2021: customizable protein-protein networks, and functional characterization of user-uploaded gene/measurement sets. Nucleic Acids Res. 49, D605–D612. 33237311 10.1093/nar/gkaa1074 PMC7779004

[44] Blake JA, Baldarelli R, Kadin JA, Richardson JE, Smith CL, and Bult CJ; Mouse Genome Database Group (2021). Mouse Genome Database (MGD): Knowledgebase for mouse-human comparative biology. Nucleic Acids Res. 49, D981–D987. 33231642 10.1093/nar/gkaa1083 PMC7779030

[45] Sinnott-Armstrong N, Tanigawa Y, Amar D, Mars N, Benner C, Aguirre M, Venkataraman GR, Wainberg M, Ollila HM, Kiiskinen T, (2021). Genetics of 35 blood and urine biomarkers in the UK Biobank. Nat. Genet. 53, 185–194. 33462484 10.1038/s41588-020-00757-z PMC7867639

[46] León S, García-Galiano D, Ruiz-Pino F, Barroso A, Manfredi-Lozano M, Romero-Ruiz A, Roa J, Vázquez MJ, Gaytan F, Blomenrohr M, (2014). Physiological roles of gonadotropin-inhibitory hormone signaling in the control of mammalian reproductive axis: studies in the NPFF1 receptor null mouse. Endocrinology 155, 2953–2965. 24823392 10.1210/en.2014-1030

[47] Weber DR, Hadjiyannakis S, McMillan HJ, Noritz G, and Ward LM (2018). Obesity and Endocrine Management of the Patient With Duchenne Muscular Dystrophy. Pediatrics 142, S43–S52. 30275248 10.1542/peds.2018-0333F PMC6460463

[48] Sakaue S, Kanai M, Tanigawa Y, Karjalainen J, Kurki M, Koshiba S, Narita A, Konuma T, Yamamoto K, Akiyama M, (2021). A cross-population atlas of genetic associations for 220 human phenotypes. Nat. Genet. 53, 1415–1424. 34594039 10.1038/s41588-021-00931-x PMC12208603

[49] Akiyama M, Okada Y, Kanai M, Takahashi A, Momozawa Y, Ikeda M, Iwata N, Ikegawa S, Hirata M, Matsuda K, (2017). Genome-wide association study identifies 112 new loci for body mass index in the Japanese population. Nat. Genet. 49, 1458–1467. 28892062 10.1038/ng.3951

[50] Beastrom N, Lu H, Macke A, Canan BD, Johnson EK, Penton CM, Kaspar BK, Rodino-Klapac LR, Zhou L, Janssen PML, and Montanaro F (2011). mdx(^5^cv) mice manifest more severe muscle dysfunction and diaphragm force deficits than do mdx Mice. Am. J. Pathol. 179, 2464–2474. 21893021 10.1016/j.ajpath.2011.07.009 PMC3204025

[51] Shpargel KB, Sengoku T, Yokoyama S, and Magnuson T (2012). UTX and UTY demonstrate histone demethylase-independent function in mouse embryonic development. PLoS Genet. 8, e1002964. 23028370 10.1371/journal.pgen.1002964 PMC3459986

[52] Thieme S, Gyárfás T, Richter C, Özhan G, Fu J, Alexopoulou D, Muders MH, Michalk I, Jakob C, Dahl A, (2013). The histone demethylase UTX regulates stem cell migration and hematopoiesis. Blood 121, 2462–2473. 23365460 10.1182/blood-2012-08-452003

[53] Zhang F, Xu L, Xu L, Xu Q, Li D, Yang Y, Karsenty G, and Chen CD (2015). JMJD3 promotes chondrocyte proliferation and hypertrophy during endochondral bone formation in mice. J. Mol. Cell Biol. 7, 23–34. 10.1093/jmcb/mjv003 PMC4342687 25587042

[54] Oh S, and Janknecht R (2012). Histone demethylase JMJD5 is essential for embryonic development. Biochem. Biophys. Res. Commun. 420, 61–65. 22402282 10.1016/j.bbrc.2012.02.115

[55] Ishimura A, Minehata K.i., Terashima M, Kondoh G, Hara T, and Suzuki T (2012). Jmjd5, an H3K36me2 histone demethylase, modulates embryonic cell proliferation through the regulation of Cdkn1a expression. Development 139, 749–759. 22241836 10.1242/dev.074138

[56] Kaelin WG (2008). Generation of a Null Allele of Rbp2, MGI Direct Data Submission to Mouse Genome Database (MGD). J:137333. https://www.informatics.jax.org/.

[57] Gracz AD, and Magness ST (2011). Sry-box (Sox) transcription factors in gastrointestinal physiology and disease. Am. J. Physiol. Gastrointest. Liver Physiol. 300, G503–G515. 21292996 10.1152/ajpgi.00489.2010 PMC3302185

[58] Iguchi H, Ikeda Y, Okamura M, Tanaka T, Urashima Y, Ohguchi H, Takayasu S, Kojima N, Iwasaki S, Ohashi R, (2005). SOX6 Attenuates Glucose-stimulated Insulin Secretion by Repressing PDX1 Transcriptional Actvity and Is Down-regulated in Hyperinsulinemic Obese Mice. J. Biol. Chem. 280, 37669–37680. 16148004 10.1074/jbc.M505392200

[59] VanHook AM (2016). SOX to be fat. Sci. Signal. 9. ec66–ec66.

[60] Pelleymounter MA, Cullen MJ, Baker MB, Hecht R, Winters D, Boone T, and Collins F (1995). Effects of the obese gene product on body weight regulation in ob/ob mice. Science 269, 540–543. 7624776 10.1126/science.7624776

[61] Campfield LA, Smith FJ, Guisez Y, Devos R, and Burn P (1995). Recombinant mouse OB protein: evidence for a peripheral signal linking adiposity and central neural networks. Science 269, 546–549. 7624778 10.1126/science.7624778

[62] Attie AD, Churchill GA, and Nadeau JH (2017). How mice are indispensable for understanding obesity and diabetes genetics. Curr. Opin. Endocrinol. Diabetes Obes. 24, 83–91. 28107248 10.1097/MED.0000000000000321 PMC5837807

[63] Doulberis M, Papaefthymiou A, Polyzos SA, Katsinelos P, Grigoriadis N, Srivastava DS, and Kountouras J (2020). Rodent models of obesity. Minerva Endocrinol. 45, 243–263. 31738033 10.23736/S0391-1977.19.03058-X

[64] Tschöp M, and Heiman ML (2002). Overview of rodent models for obesity research. Curr. Protoc. Neurosci. Chapter 9, Unit 9.10. 10.1002/0471142301.ns0910s17 18428569

[65] Kanasaki K, and Koya D (2011). Biology of obesity: lessons from animal models of obesity. J. Biomed. Biotechnol. 2011, 197636. 21274264 10.1155/2011/197636 PMC3022217

[66] Crouse WL, Das SK, Le T, Keele G, Holl K, Seshie O, Craddock AL, Sharma NK, Comeau ME, Langefeld CD, (2022). Transcriptome-wide analyses of adipose tissue in outbred rats reveal genetic regulatory mechanisms relevant for human obesity. Physiol. Genom. 54, 206–219. 10.1152/physiolgenomics.00172.2021 PMC9142160 35467982

[67] Linke V, Overmyer KA, Miller IJ, Brademan DR, Hutchins PD, Trujillo EA, Reddy TR, Russell JD, Cushing EM, Schueler KL, (2020). A large-scale genome-lipid association map guides lipid identification. Nat. Metab. 2, 1149–1162. 32958938 10.1038/s42255-020-00278-3 PMC7572687

[68] Martin AR, Kanai M, Kamatani Y, Okada Y, Neale BM, and Daly MJ (2019). Clinical use of current polygenic risk scores may exacerbate health disparities. Nat. Genet. 51, 584–591. 30926966 10.1038/s41588-019-0379-x PMC6563838

[69] Miller GD (2019). Appetite Regulation: Hormones, Peptides, and Neurotransmitters and Their Role in Obesity. Am. J. Lifestyle Med. 13, 586–601. 31662725 10.1177/1559827617716376 PMC6796227

[70] Qaid MM, and Abdelrahman MM (2016). Role of insulin and other related hormones in energy metabolism—A review. Cogent Food Agric. 2, 1267691.

[71] Mul JD, van Boxtel R, Bergen DJM, Brans MAD, Brakkee JH, Toonen PW, Garner KM, Adan RAH, and Cuppen E (2012). Melanocortin receptor 4 deficiency affects body weight regulation, grooming behavior, and substrate preference in the rat. Obesity 20, 612–621. 21527895 10.1038/oby.2011.81 PMC3286758

[72] Ashburner M, Ball CA, Blake JA, Botstein D, Butler H, Cherry JM, Davis AP, Dolinski K, Dwight SS, Eppig JT, (2000). Gene ontology: tool for the unification of biology. The Gene Ontology Consortium. Nat. Genet. 25, 25–29. 10802651 10.1038/75556 PMC3037419

[73] Gene Ontology Consortium (2021). The Gene Ontology resource: enriching a GOld mine. Nucleic Acids Res. 49, D325–D334. 33290552 10.1093/nar/gkaa1113 PMC7779012

[74] Backman JD, Li AH, Marcketta A, Sun D, Mbatchou J, Kessler MD, Benner C, Liu D, Locke AE, Balasubramanian S, (2021). Exome sequencing and analysis of 454,787 UK Biobank participants. Nature 599, 628–634. 34662886 10.1038/s41586-021-04103-z PMC8596853

[75] Nasser J, Bergman DT, Fulco CP, Guckelberger P, Doughty BR, Patwardhan TA, Jones TR, Nguyen TH, Ulirsch JC, Lekschas F, (2021). Genome-wide enhancer maps link risk variants to disease genes. Nature 593, 238–243. 33828297 10.1038/s41586-021-03446-x PMC9153265

[76] Pietzner M, Wheeler E, Carrasco-Zanini J, Cortes A, Koprulu M, Wörheide MA, Oerton E, Cook J, Stewart ID, Kerrison ND, (2021). Mapping the proteo-genomic convergence of human diseases. Science 374, eabj1541. 34648354 10.1126/science.abj1541 PMC9904207

[77] Skinnider MA, Scott NE, Prudova A, Kerr CH, Stoynov N, Stacey RG, Chan QWT, Rattray D, Gsponer J, and Foster LJ (2021). An atlas of protein-protein interactions across mouse tissues. Cell 184, 4073–4089.e17. 34214469 10.1016/j.cell.2021.06.003

[78] Ha D, Kim D, Kim I, Oh Y, Kong J, Han SK, and Kim S (2022). Evolutionary rewiring of regulatory networks contributes to phenotypic differences between human and mouse orthologous genes. Nucleic Acids Res. 50, 1849–1863. 35137181 10.1093/nar/gkac050 PMC8887464

[79] Alanis-Lobato G, Möllmann JS, Schaefer MH, and Andrade-Navarro MA (2020). the mouse integrated protein-protein interaction reference. Database 2020, baaa035. 32496562 10.1093/database/baaa035 PMC7271249

[80] Tao Y-T, Ding X-B, Jin J, Zhang H-B, Guo W-P, Ruan L, Yang Q-L, Chen P-C, Yao H, and Chen X (2020). Predicted rat interactome database and gene set linkage analysis. Database 2020. 10.1093/database/baaa086. PMC7678787 33216897

[81] Chen Y, Liu Y, Du M, Zhang W, Xu L, Gao X, Zhang L, Gao H, Xu L, Li J, and Zhao M (2017). Constructing a comprehensive gene co-expression based interactome in Bos taurus. PeerJ 5, e4107. 29226034 10.7717/peerj.4107 PMC5719962

[82] Bossi A, and Lehner B (2009). Tissue specificity and the human protein interaction network. Mol. Syst. Biol. 5, 260. 19357639 10.1038/msb.2009.17 PMC2683721

[83] Huttlin EL, Bruckner RJ, Navarrete-Perea J, Cannon JR, Baltier K, Gebreab F, Gygi MP, Thornock A, Zarraga G, Tam S, (2021). Dual proteome-scale networks reveal cell-specific remodeling of the human interactome. Cell 184, 3022–3040.e28. 33961781 10.1016/j.cell.2021.04.011 PMC8165030

[84] Smith CL, and Eppig JT (2009). The mammalian phenotype ontology: enabling robust annotation and comparative analysis. Wiley Interdiscip. Rev. Syst. Biol. Med. 1, 390–399. 20052305 10.1002/wsbm.44 PMC2801442

[85] Babraham Bioinformatics. FastQC (2017). A Quality Control Tool for High Throughput Sequence Data (Cambridge, UK: Babraham Institute).

[86] Li H, and Durbin R (2009). Fast and accurate short read alignment with Burrows-Wheeler transform. Bioinformatics 25, 1754–1760. 19451168 10.1093/bioinformatics/btp324 PMC2705234

[87] McKenna A, Hanna M, Banks E, Sivachenko A, Cibulskis K, Kernytsky A, Garimella K, Altshuler D, Gabriel S, Daly M, and DePristo MA (2010). The Genome Analysis Toolkit: a MapReduce framework for analyzing next-generation DNA sequencing data. Genome Res. 20, 1297–1303. 20644199 10.1101/gr.107524.110 PMC2928508

[88] Korneliussen TS, Albrechtsen A, and Nielsen R (2014). Analysis of Next Generation Sequencing Data. BMC Bioinf. 15, 356. 10.1186/s12859-014-0356-4 PMC4248462 25420514

[89] Durvasula A, Hoffman PJ, Kent TV, Liu C, Kono TJY, Morrell PL, and Ross-Ibarra J (2016). angsd-wrapper: utilities for analysing next-generation sequencing data. Mol. Ecol. Resour. 16, 1449–1454. 27480660 10.1111/1755-0998.12578

[90] Purcell S, Neale B, Todd-Brown K, Thomas L, Ferreira MAR, Bender D, Maller J, Sklar P, de Bakker PIW, Daly MJ, and Sham PC (2007). PLINK: a tool set for whole-genome association and population-based linkage analyses. Am. J. Hum. Genet. 81, 559–575. 17701901 10.1086/519795 PMC1950838

[91] Chang CC, Chow CC, Tellier LC, Vattikuti S, Purcell SM, and Lee JJ (2015). Second-generation PLINK: rising to the challenge of larger and richer datasets. GigaScience 4, 7. 25722852 10.1186/s13742-015-0047-8 PMC4342193

[92] Browning BL, and Browning SR (2016). Genotype Imputation with Millions of Reference Samples. Am. J. Hum. Genet. 98, 116–126. 26748515 10.1016/j.ajhg.2015.11.020 PMC4716681

[93] Howie BN, Donnelly P, and Marchini J (2009). A flexible and accurate genotype imputation method for the next generation of genome-wide association studies. PLoS Genet. 5, e1000529. 19543373 10.1371/journal.pgen.1000529 PMC2689936

[94] Yang J, Hong Lee S, Goddard ME, and Visscher PM (2011). GCTA: A Tool for Genome-wide Complex Trait Analysis. Am. J. Hum. Genet. 88, 76–82. 10.1016/j.ajhg.2010.11.011. 21167468 PMC3014363

[95] Lamparter D, Marbach D, Rueedi R, Kutalik Z, and Bergmann S (2016). Fast and Rigorous Computation of Gene and Pathway Scores from SNP-Based Summary Statistics. PLoS Comput. Biol. 12, e1004714. 26808494 10.1371/journal.pcbi.1004714 PMC4726509

[96] Zheng F, Zhang S, Churas C, Pratt D, Bahar I, and Ideker T (2021). HiDeF: identifying persistent structures in multiscale ‘omics data. Genome Biol. 22, 21,. Preprint at. 10.1186/s13059-020-02228-4. 33413539 PMC7789082

[97] Raudvere U, Kolberg L, Kuzmin I, Arak T, Adler P, Peterson H, and Vilo J (2019). g:Profiler: a web server for functional enrichment analysis and conversions of gene lists (2019 update). Nucleic Acids Res. 47, W191–W198. 31066453 10.1093/nar/gkz369 PMC6602461

[98] Dougherty JD, Schmidt EF, Nakajima M, and Heintz N (2010). Analytical approaches to RNA profiling data for the identification of genes enriched in specific cells. Nucleic Acids Res. 38, 4218–4230. 20308160 10.1093/nar/gkq130 PMC2910036

[99] Jain A, and Tuteja G (2019). TissueEnrich: Tissue-specific gene enrichment analysis. Bioinformatics 35, 1966–1967. 30346488 10.1093/bioinformatics/bty890 PMC6546155

[100] Shannon P, Markiel A, Ozier O, Baliga NS, Wang JT, Ramage D, Amin N, Schwikowski B, and Ideker T (2003). Cytoscape: a software environment for integrated models of biomolecular interaction networks. Genome Res. 13, 2498–2504. 14597658 10.1101/gr.1239303 PMC403769

[101] Loos RJ (2018). The genetics of adiposity. Curr. Opin. Genet. Dev. 50, 86–95. 29529423 10.1016/j.gde.2018.02.009 PMC6089650

[102] Eyre TA, Wright MW, Lush MJ, and Bruford EA (2007). HCOP: a searchable database of human orthology predictions. Briefings Bioinf. 8, 2–5. 10.1093/bib/bbl030 16951416

[103] Yates B, Gray KA, Jones TEM, and Bruford EA (2021). Updates to HCOP: the HGNC comparison of orthology predictions tool. Briefings Bioinf. 22, bbab155. 10.1093/bib/bbab155 PMC8574622 33959747

[104] Hermsen R, de Ligt J, Spee W, Blokzijl F, Schäfer S, Adami E, Boymans S, Flink S, van Boxtel R, van der Weide RH, (2015). Genomic landscape of rat strain and substrain variation. BMC Genom. 16, 357. 10.1186/s12864-015-1594-1 PMC4422378 25943489

[105] Ramdas S, Ozel AB, Treutelaar MK, Holl K, Mandel M, Woods LCS, and Li JZ (2019). Extended regions of suspected mis-assembly in the rat reference genome. Sci. Data 6, 39. 31015470 10.1038/s41597-019-0041-6 PMC6478900

[106] Gileta AF, Gao J, Chitre AS, Bimschleger HV, St Pierre CL, Gopalakrishnan S, and Palmer AA (2020). Adapting Genotyping-by-Sequencing and Variant Calling for Heterogeneous Stock Rats. G3 10, 2195–2205. 32398234 10.1534/g3.120.401325 PMC7341140

[107] Cheng R, Parker CC, Abney M, and Palmer AA (2013). Practical considerations regarding the use of genotype and pedigree data to model relatedness in the context of genome-wide association studies. G3 3, 1861–1867. 23979941 10.1534/g3.113.007948 PMC3789811

[108] Gonzales NM, Seo J, Hernandez Cordero AI, St Pierre CL, Gregory JS, Distler MG, Abney M, Canzar S, Lionikas A, and Palmer AA (2018). Genome wide association analysis in a mouse advanced intercross line. Nat. Commun. 9, 5162. 30514929 10.1038/s41467-018-07642-8 PMC6279738

[109] 1000 Genomes Project Consortium; Auton A, Brooks LD, Durbin RM, Garrison EP, Kang HM, Korbel JO, Marchini JL, McCarthy S, McVean GA, and Abecasis GR (2015). A global reference for human genetic variation. Nature 526, 68–74. 26432245 10.1038/nature15393 PMC4750478

[110] Davies RB (1980). Algorithm AS 155: The Distribution of a Linear Combination of χ^2^ Random Variables. Appl. Stat. 29, 323–333.

[111] Farebrother RW, and Algorithm AS (1984). The Distribution of a Positive Linear Combination of χ^2^ Random Variables. Appl. Stat. 33, 332–339. 204.

[112] Guney E, Menche J, Vidal M, and Barábasi AL (2016). Network-based in silico drug efficacy screening. Nat. Commun. 7, 10331. 26831545 10.1038/ncomms10331 PMC4740350

[113] Singhal A, Cao S, Churas C, Pratt D, Fortunato S, Zheng F, and Ideker T (2020). Multiscale community detection in Cytoscape. PLoS Comput. Biol. 16, e1008239. 33095781 10.1371/journal.pcbi.1008239 PMC7584444

[114] Bult CJ, Blake JA, Smith CL, Kadin JA, and Richardson JE; Mouse Genome Database Group (2019). Mouse Genome Database (MGD) 2019. Nucleic Acids Res. 47, D801–D806. 30407599 10.1093/nar/gky1056 PMC6323923

[115] GTEx Consortium (2013). The Genotype-Tissue Expression (GTEx) project. Nat. Genet. 45, 580–585. 23715323 10.1038/ng.2653 PMC4010069

[116] Shen Y, Yue F, McCleary DF, Ye Z, Edsall L, Kuan S, Wagner U, Dixon J, Lee L, Lobanenkov VV, and Ren B (2012). A map of the cisregulatory sequences in the mouse genome. Nature 488, 116–120. 22763441 10.1038/nature11243 PMC4041622

