# Genome-wide association studies of human and rat BMI converge on synapse, epigenome, and hormone signaling networks

**DOI:** 10.1016/j.celrep.2025.116044

**Published:** 2025-07-16

**Authors:** Sarah N. Wright, Brittany S. Leger, Sara Brin Rosenthal, Sophie N. Liu, Tongqiu Jia, Apurva S. Chitre, Oksana Polesskaya, Katie Holl, Jianjun Gao, Riyan Cheng, Angel Garcia Martinez, Anthony George, Alexander F. Gileta, Wenyan Han, Alesa H. Netzley, Christopher P. King, Alexander Lamparelli, Connor Martin, Celine L. St. Pierre, Tengfei Wang, Hannah Bimschleger, Jerry Richards, Keita Ishiwari, Hao Chen, Shelly B. Flagel, Paul Meyer, Terry E. Robinson, Leah C. Solberg Woods, Jason F. Kreisberg, Trey Ideker, Abraham A. Palmer

The original ENA upload was missing the majority of the genotypes for the paper’s dataset. This has been corrected with a new upload to ENA, so the ENA Analysis access code that appears in the key resources table and the data and code availability section of the published paper is no longer accurate. The correct accession number is ERZ25071606. The authors regret this oversight.

