# Supplementary material for: Genome-wide association studies of human and rat BMI converge on synapse, epigenome, and hormone signaling networks
Source: Cell Rep. Author manuscript; Available in PMC 2023 Oct 3. (PMC10546330; doi:10.1016/j.celrep.2023.112873)
Supplement: 1 [file NIHMS1928248-supplement-1.pdf]

Cell Reports, Volume *42*

Supplemental information

Genome-wide association studies of human and rat BMI converge on synapse, epigenome, and hormone signaling networks

Sarah N. Wright, Brittany S. Leger, Sara Brin Rosenthal, Sophie N. Liu, Tongqiu Jia, Apurva S. Chitre, Oksana Polesskaya, Katie Holl, Jianjun Gao, Riyan Cheng, Angel Garcia Martinez, Anthony George, Alexander F. Gileta, Wenyan Han, Alesa H. Netzley, Christopher P. King, Alexander Lamparelli, Connor Martin, Celine L. St. Pierre, Tengfei Wang, Hannah Bimschleger, Jerry Richards, Keita Ishiwari, Hao Chen, Shelly B. Flagel, Paul Meyer, Terry E. Robinson, Leah C. Solberg Woods, Jason F. Kreisberg, Trey Ideker, and Abraham A. Palmer

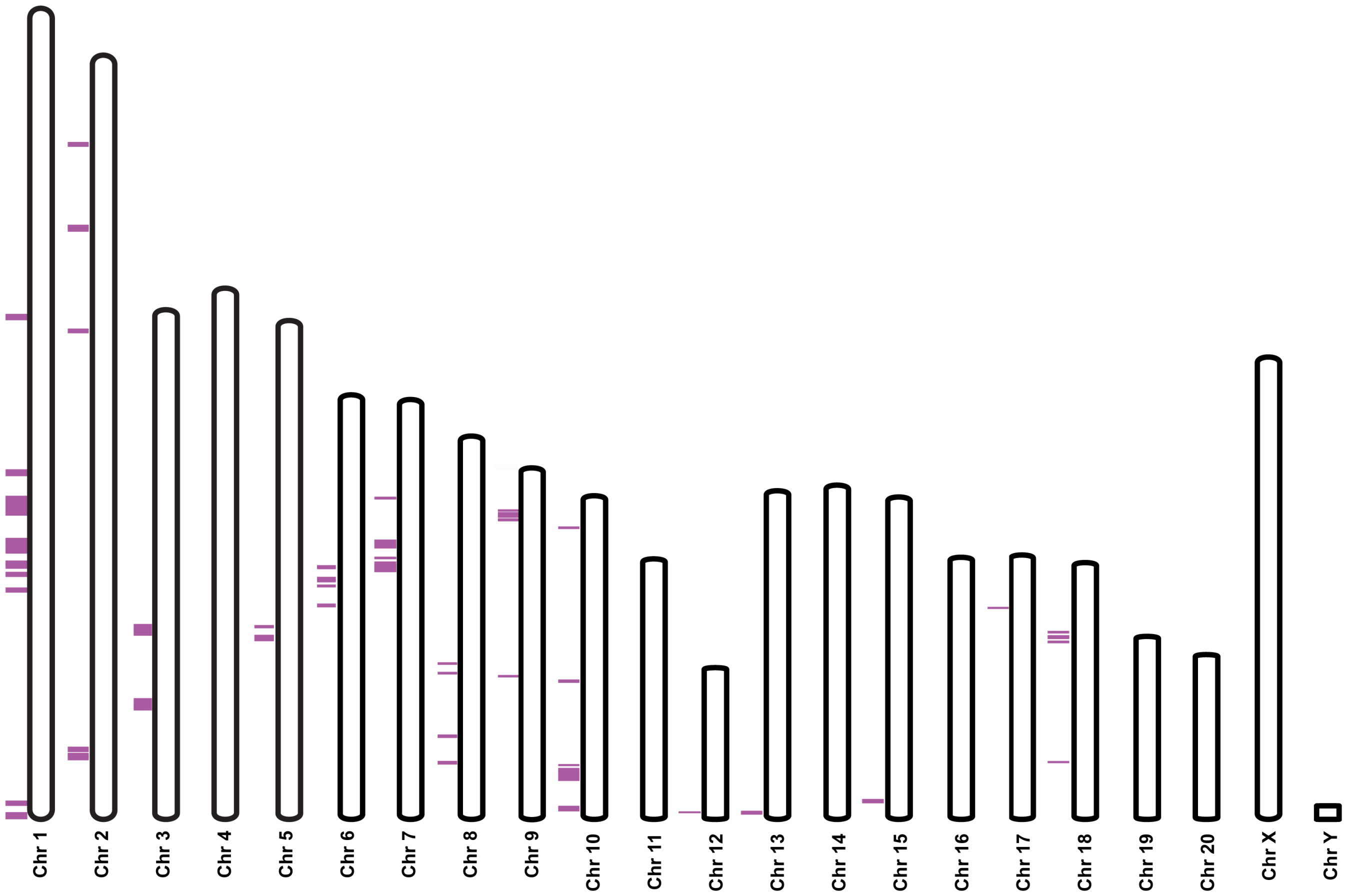

Figure S1. Positions of Rat BMI seed genes in the rat genome, related to STAR Methods. Genomic regions of rat BMI seed genes, including a 10kb buffer around genes, indicated as purple bars annotated on rat chromosomes. Chromosome number is indicated below each chromosome.

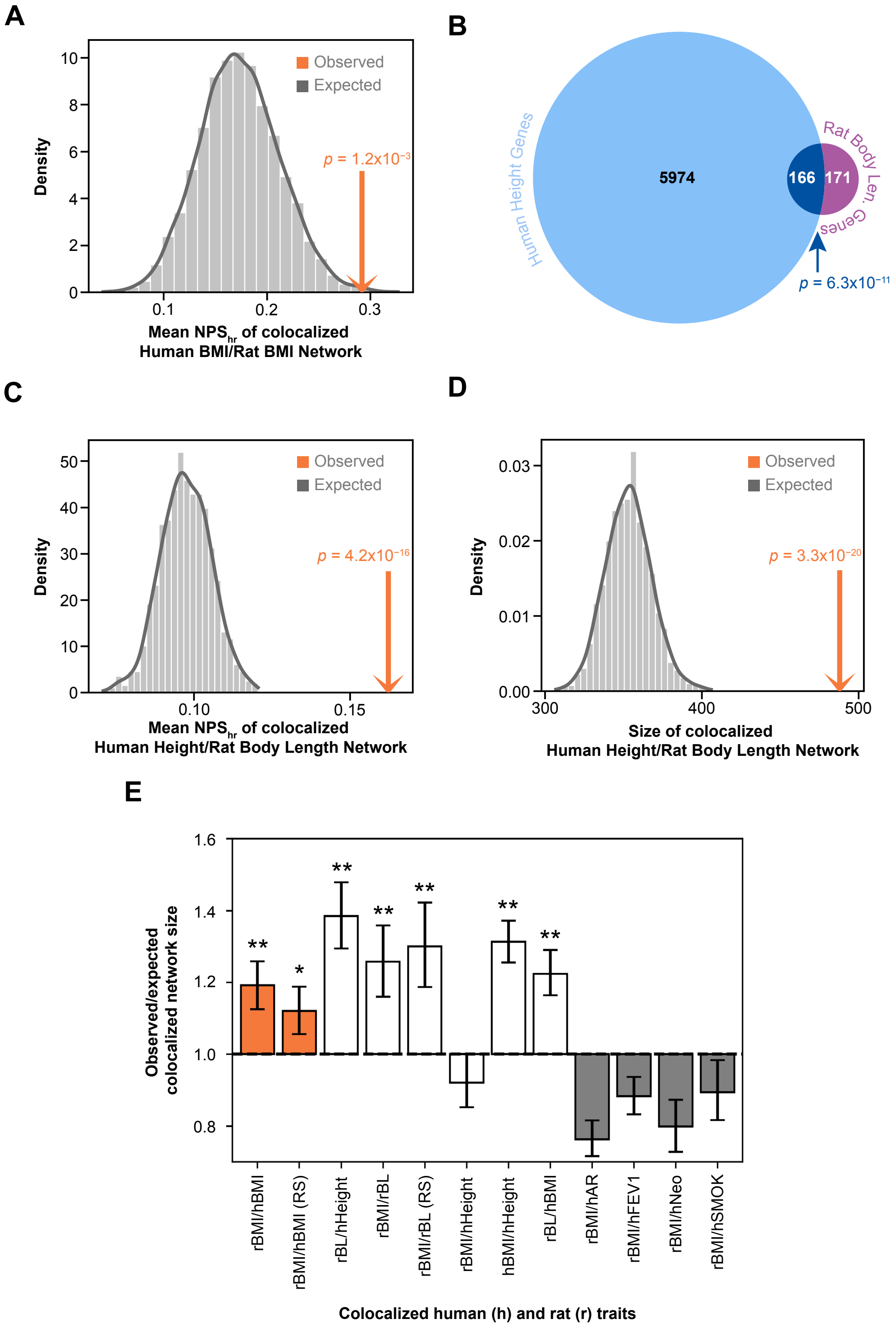

Figure S2. Network colocalization statistics for human and rat BMI and height/body length, Related to Figure 2. (A) Observed (orange arrow) and expected mean NPS_{hr} for colocalization of human and rat BMI genes, with significance assessed by Z-test. (B) Overlap of input gene sets for human height and rat body length (BL) (hypergeometric test). (C) Observed (orange arrow) and expected mean NPS_{hr} for human height and rat BL, with significance assessed by Z-test. (D) Observed (orange arrow) and expected size of colocalized network for human height and rat BL using the same thresholds as for the BMI study ($NPS_h > 1$, $NPS_r > 1$ and $NPS_{hr} > 3$), with significance assessed by Z-test. (E) Observed over expected size of the colocalized network for human (h) and rat (r), BMI (orange), body length (BL)/height (white), and negative control (gray) comparisons. FEV1: forced expiratory volume per second, AR: allergic rhinitis symptoms, SMOK: never smoked cigarettes, Neo: non-cancer neoplasms. All comparisons utilized PCNet as the interactome, except for those indicated as RS, which utilized the high confidence rat STRING network. Vertical bars indicate 95% confidence intervals. Significance calculated by Z-test, Bonferroni corrected (* $p < 5x10{-}4$, ** $p < 1x10{-}7$).

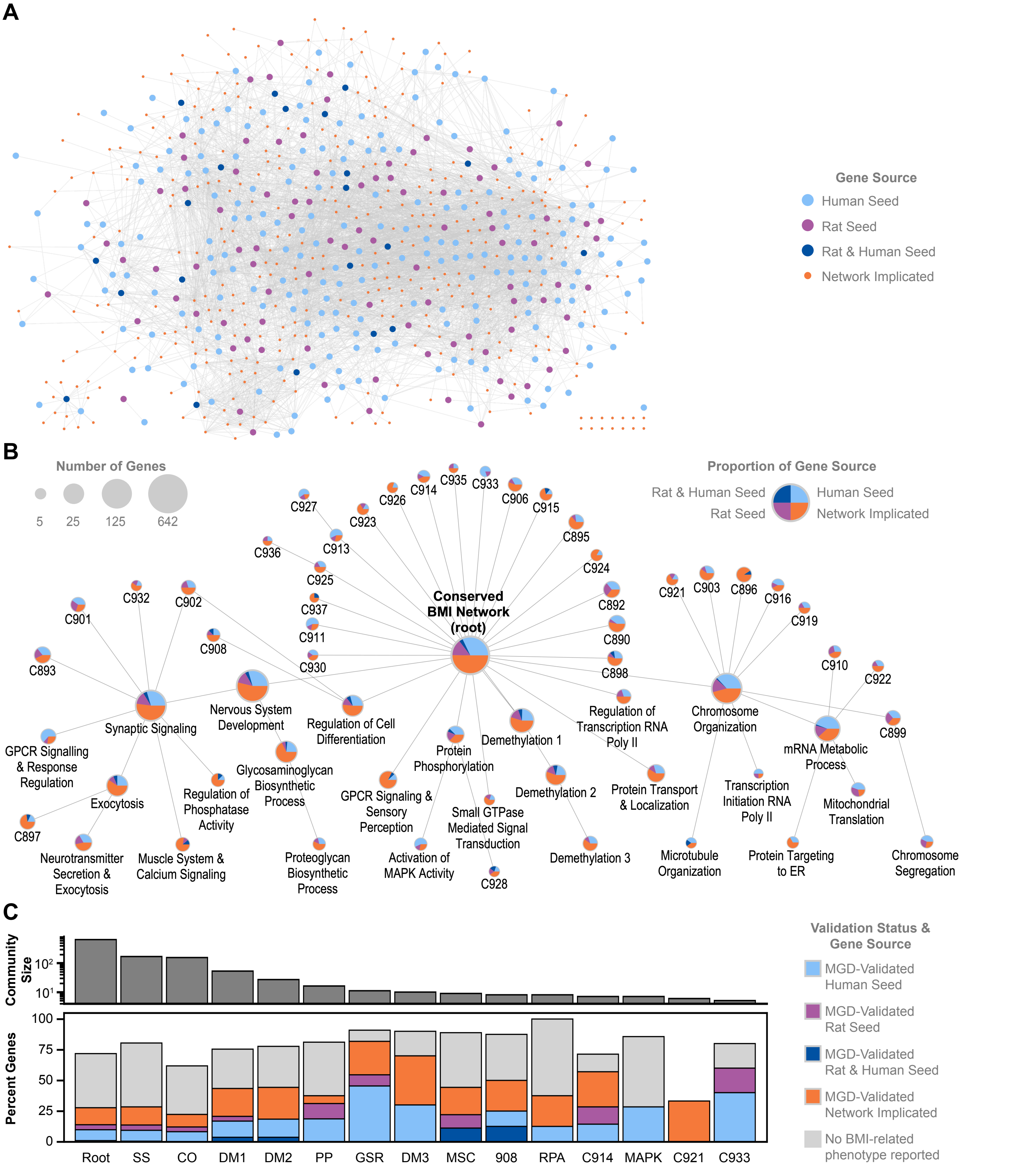

Figure S3: The conserved BMI network and complete systems map, Related to Figures 2 and 3. (A) Subnetwork of PCNet including all genes proximal to both human and rat BMI seed genes. Light blue nodes indicate human seed genes, purple nodes indicate rat seed genes, dark blue nodes indicate seeds in both species, and orange nodes are network-implicated genes. Edges maintained from PCNet. (B) Conserved BMI systems map, including annotated and unannotated communities. Systems are annotated based on enriched GO biological process terms (where available) or a unique system identifier. Node size represents the number of genes per system, and node pie chart represents the source of genes within the system. (C) Sources of all MGD-validated genes for communities significantly enriched for body-size associated genes (bottom). MGD-validated genes were defined as genes linked to at least one BMI-related phenotype in MGD. Total height of bar represents the proportion of genes reported in the MGD database. Number of genes in each community (top).

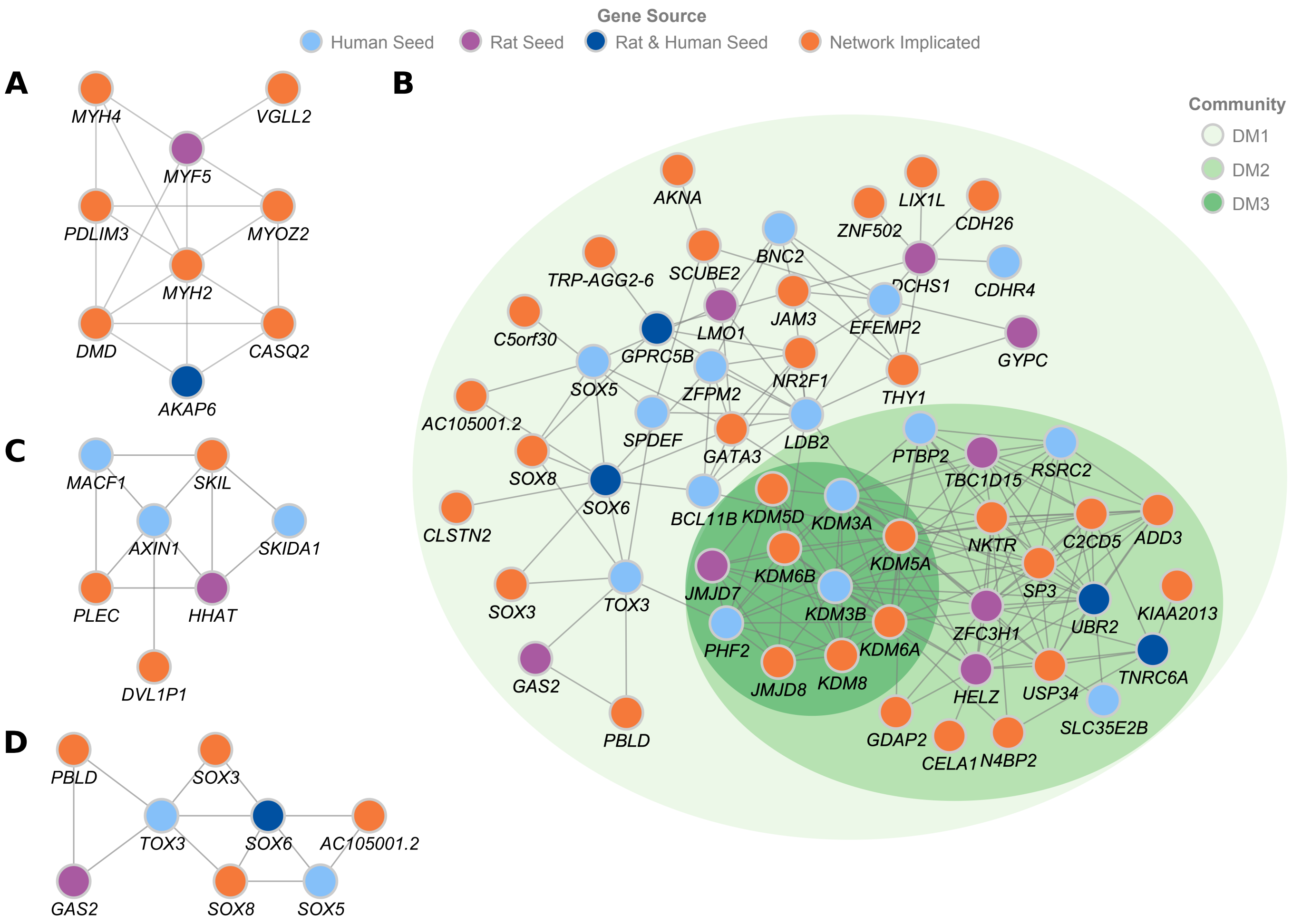

Figure S4: Subnetworks for select communities from the BMI systems map enriched for body-size associated genes, Related to Figures 3 and 5. Node color indicates the source of genes as human seed genes (light blue), rat seed genes (purple), human & rat seed genes (dark blue), or network-implicated (orange). Color scheme used in all panels. (A) Muscle System & Calcium Signaling (MSC).B) Nested demethylation communities Demethylation 1 (DM1, outer), Demethylation 2 (DM2, middle), Demethylation 3 (DM3, inner). (C) Unannotated community C914. (D) Unannotated community C908.

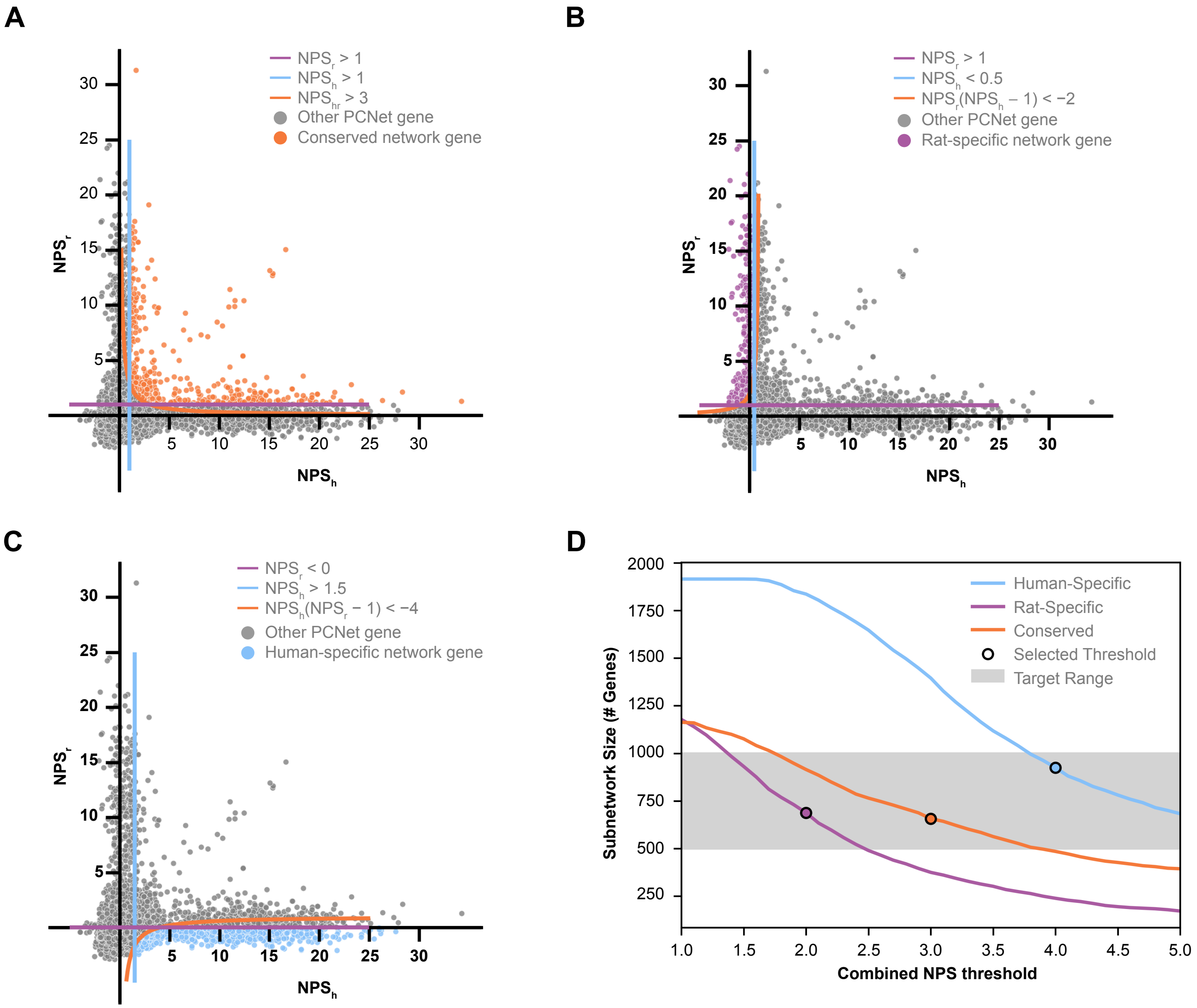

Figure S5: Definition of the conserved and cross-species BMI networks from network proximity scores, Related to Figure 6 and STAR Methods. Thresholds are defined on NPS_h, NPS_r, and NPS_{hr} to define the set of genes in the (A) conserved, (B) rat-specific, and (C) human-specific BMI subnetworks. (D) Size of species-specific and conserved subnetworks at varying combined NPS thresholds. Black circles indicate the size of the respective networks at the thresholds selected for this study. Gray background indicates the target range for the network sizes.

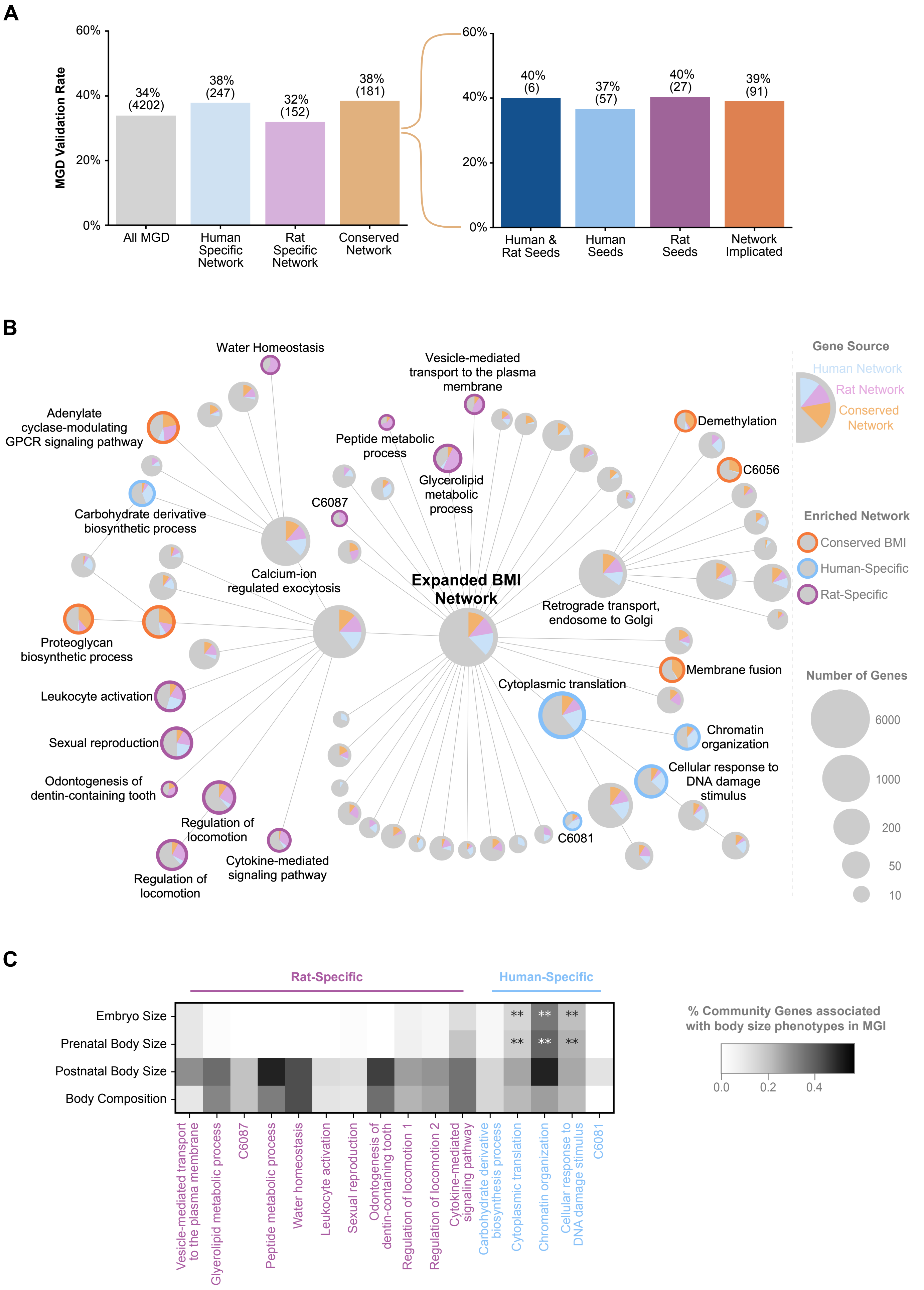
A
MGD Validation Rate
60%
40%
20%
0%
34%
(4202)
38%
(247)
32%
(152)
38%
(181)
All MGD
Human Specific Network
Rat Specific Network
Conserved Network
40%
(6)
37%
(57)
40%
(27)
39%
(91)
Human & Rat Seeds
Human Seeds
Rat Seeds
Network Implicated
B
Expanded BMI Network
Water Homeostasis
Vesicle-mediated transport to the plasma membrane
Adenylate cyclase-modulating GPCR signaling pathway
Peptide metabolic process
Glycerolipid metabolic process
Demethylation
C6056
C6087
Carbohydrate derivative biosynthetic process
Calcium-ion regulated exocytosis
Retrograde transport, endosome to Golgi
Proteoglycan biosynthetic process
Membrane fusion
Cytoplasmic translation
Leukocyte activation
Sexual reproduction
Chromatin organization
Cellular response to DNA damage stimulus
Odontogenesis of dentin-containing tooth
Regulation of locomotion
Cytokine-mediated signaling pathway
Regulation of locomotion
C6081
Gene Source
Human Network
Rat Network
Conserved Network
Enriched Network
Conserved BMI
Human-Specific
Rat-Specific
Number of Genes
6000
1000
200
50
10
C
Rat-Specific
Human-Specific
Embryo Size
Prenatal Body Size
Postnatal Body Size
Body Composition
**
Vesicle-mediated transport to the plasma membrane
Glyerolipid metabolic process
C6087
Peptide metabolic process
Water homeostasis
Leukocyte activation
Sexual reproduction
Odontogenesis of dentin-containing tooth
Regulation of locomotion 1
Regulation of locomotion 2
Cytokine-mediated signaling pathway
Carbohydrate derivative biosynthesis process
Cytoplasmic translation
Chromatin organization
Cellular response to DNA damage stimulus
C6081
% Community Genes associated with body size phenotypes in MGI
0.0
0.2
0.4

Figure S6. Species-specific network statistics and the full expanded BMI systems map, Related to Figure 6. (A) Proportion of genes in the species-specific and conserved networks associated with body size phenotypes in MGD (left), calculated as percent of network genes that have been assessed phenotypically by MGD. Right shows the proportion of genes in the conserved network associated with body size phenotypes in mice, broken down by gene source. (B) The expanded systems map including all annotated and unannotated communities. Node size indicates the number of genes in each community and pie charts show the fraction genes from each of the human-specific, rat-specific, and conserved networks. Highlighted communities are those enriched for genes in one of these three subnetworks ($q < 0.05$, hypergeometric test). Enriched communities are annotated based on associated GO Biological Process terms (where available) or a unique system identifier. (C) Proportion of community genes associated with BMI-relevant phenotypes in mice, for all human and rat specific communities. Label color indicates the community as rat-specific (purple) or human-specific (blue). Proportion is calculated as a percent of network genes that have been assessed phenotypically by MGD. ** indicates a BH-correct p value < 0.05 via hypergeometric test.
